# Effectiveness of physical therapy interventions for children with cerebral palsy: A systematic review

**DOI:** 10.1186/1471-2431-8-14

**Published:** 2008-04-24

**Authors:** Heidi Anttila, Ilona Autti-Rämö, Jutta Suoranta, Marjukka Mäkelä, Antti Malmivaara

**Affiliations:** 1 Finnish Office for Health Technology Assessment (FinOHTA), at the National Research and Development Centre for Welfare and Health (STAKES), PO Box 220, FIN-00531 Helsinki, Finland; 2 The Social Insurance Institute, PO Box 450, 00101 Helsinki, Finland; 3 Hospital of Children and Adolescents, University of Helsinki, PO Box 280, 00029 HUS, Finland; 4 Tampere School of Public Health, 33014 University of Tampere, Finland; 5 Department of General Practice, University of Copenhagen, PO Box 2099, 1014 Copenhagen K, Denmark

## Abstract

**Background:**

To assess the effectiveness of physical therapy (PT) interventions on functioning in children with cerebral palsy (CP).

**Methods:**

A search was made in Medline, Cinahl, PEDro and the Cochrane library for the period 1990 to February 2007. Only randomized controlled trials (RCTs) on PT interventions in children with diagnosed CP were included. Two reviewers independently assessed the methodological quality and extracted the data. The outcomes measured in the trials were classified using the International Classification of Functioning, Disability and Health (ICF).

**Results:**

Twenty-two trials were identified. Eight intervention categories were distinguished. Four trials were of high methodological quality. Moderate evidence of effectiveness was established for two intervention categories: effectiveness of upper extremity treatments on attained goals and active supination, and of prehensile hand treatment and neurodevelopmental therapy (NDT) or NDT twice a week on developmental status, and of constraint-induced therapy on amount and quality of hand use. Moderate evidence of ineffectiveness was found of strength training on walking speed and stride length. Conflicting evidence was found for strength training on gross motor function. For the other intervention categories the evidence was limited due to low methodological quality and the statistically insignificant results of the studies.

**Conclusion:**

Due to limitations in methodological quality and variations in population, interventions and outcomes, mostly limited evidence on the effectiveness of most PT interventions is available through RCTs. Moderate evidence was found for some effectiveness of upper extremity training. Well-designed trials are needed especially for focused PT interventions.

## Background

Cerebral palsy (CP) describes "a group of permanent disorders of the development of movement and posture, causing activity limitation, that are attributed to nonprogressive disturbances that occurred in the developing fetal or infant brain. The motor disorders of cerebral palsy are often accompanied by disturbances of sensation, perception, cognition, communication, and behaviour, by epilepsy, and by secondary musculoskeletal problems [1]." The estimated prevalence in the general population is 2/1000 [2,3]. The limitations in activity require individual rehabilitation throughout life [4].

Physical therapy (PT) plays a central role in managing the condition; it focuses on function, movement, and optimal use of the child's potential. PT uses physical approaches to promote, maintain and restore physical, psychological and social well-being. Physiotherapists also teach parents how to handle their child at home for feeding, bathing, dressing and other activities, and give advice on mobility devices [5,6].

Physiotherapists emphasize the need for the practice to be evidence based whenever possible [5]. Previous reviews have addressed the effectiveness of PT interventions for children with CP focusing on neurodevelopmental therapy (NDT) [7-9], strength training [10,11], conductive education [12-15], various PT interventions [16-19], or orthotic devices [20,21]. These systematic reviews covered various study designs, with only a few assessing the study quality, and only 12 randomized controlled trials (RCTs) were identified between 1973 and 1998. More recent systematic review topics included focused interventions, such as constrained-induced movement therapy [22], postural control [23], passive stretching [24], hydrotherapy [25], hippotherapy [26,27], and orthotic devices [28]. Overall, the effectiveness and efficacy of therapeutic interventions for children with CP has been difficult to determine owing to the lack of high-quality research. Siebes et al [29] identified an improvement in the methodological quality of the therapeutic intervention studies during the last decade, and Kunz et al [30] found the quality of PT trials to be better than their reputation.

Therapists, doctors and parents need new knowledge of the effects of widely used PT interventions for evidence-based decision-making. We wanted to evaluate the effectiveness of interventions in current use, i.e. published since 1990, as established in well-designed randomized studies.

## Methods

### Literature searches

We searched Medline, the Physiotherapy Evidence Database PEDro [31], CINAHL (a database for allied health and nursing), and the Cochrane Controlled Trials Register from 1990 to February 2007. The reference lists of the identified studies and reviews were screened for additional references. An experienced medical librarian formulated the search strategy for Ovid Medline (see Additional file 1, Word document: search strategy for Ovid Medline) and adapted it to the other databases.

### Inclusion criteria

#### Study type

Published, full-length articles or full written reports of RCTs since 1990.

#### Population

Participants had to be children or adolescents with diagnosed CP and aged three months to 20 years at the start of the program. If more than 20% of the study population consisted of other conditions or exceeded the age limits and the data could not be separated, the study was excluded.

#### Interventions

Studies using clinically justifiable PT interventions, or a combination of these, as compared to placebo, sham therapy, or other PT interventions were included. Methods such as biofeedback and electrical stimulation, or behavioral or educational approaches such as conductive education, were not included as main therapies but were accepted as an adjunct therapy if given to all study groups. Trials providing other adjuncts to PT, such as selective dorsal rhizotomy, botulinum injection therapy, or intrathecal baclofen were excluded. In addition, studies on surgical or pharmaceutical interventions, dental care, oral motor control (drooling, swallowing, speech and communication), nutrition, acupuncture, psychotherapy, and hyperbaric oxygen therapy were excluded.

#### Outcomes

Any components of functioning or disability according to the International Classification of Functioning, Disability and Health (ICF) [32].

#### Language

Danish, English, Finnish, French, German, Norwegian and Swedish.

### Study selection, data extraction and assessment of the methodological quality

Two reviewers (HA and IAR) independently screened the search results and selected articles for closer scrutiny. After full texts were ordered, two reviewers (HA and IAR) separately assessed them for inclusion criteria.

Two reviewers (HA and IAR or JS) assessed the quality of the trials using criteria and decision rules modified from Van Tulder et al [33] (see Additional file 2, Word document: Quality assessment criteria and decision rules). These include internal validity criteria (n = 11) related to selection bias (criteria a and b), performance bias (criteria d, e, g, and h), attrition bias (criteria i and k) and detection bias (criteria f and j). All items were rated as "yes", "no" or "don't know". We counted a summary score for "yes" answers and considered studies as of high quality if they had adequate randomization and group allocation concealment, similar prognostic factors at baseline and a described and acceptable drop-out rate. A third reviewer (IAR or AM) checked the quality assessment in cases of disagreement.

Two reviewers (HA and IAR or JS) extracted data on patients, interventions and outcomes. The feasibility of the data extraction form (see Additional file 3, Word document: data extraction form) was tested with a sample of three articles eligible for this review.

### Data synthesis methods

The diversity among studies with regard to patients (type and severity of CP), interventions (type, frequency, duration, and setting), outcome measures (diversity, presentation of the results), and methodological quality of the studies did not allow us to perform a quantitative analysis (meta-analysis). For a qualitative summary, the interventions were grouped and analyzed separately for each intervention category. The outcomes were divided to ICF components (body functions and structures, activities and participation, environmental factors and personal factors) according to the major focus of measurement. The results for all outcomes of each trial were grouped according to the presence of statistically significant differences between groups: 1) difference in favor of the intervention group 2) difference in favor of the control group, 3) no difference, 4) not analyzed. The levels of evidence synthesis used in this review is based on the method by van Tulder et al [33] (Table 1).

**Table 1:** Levels of evidence (adapted and modified from van Tulder et al [33])

Strong	Consistent findings among multiple high-quality RCTs
Moderate	Consistent findings among multiple low-quality RCTs and/or one high-quality RCT
Limited	One low-quality RCT
Conflicting	Inconsistent findings among multiple trials
No evidence	No RCTs

## Results

**Figure 1:**
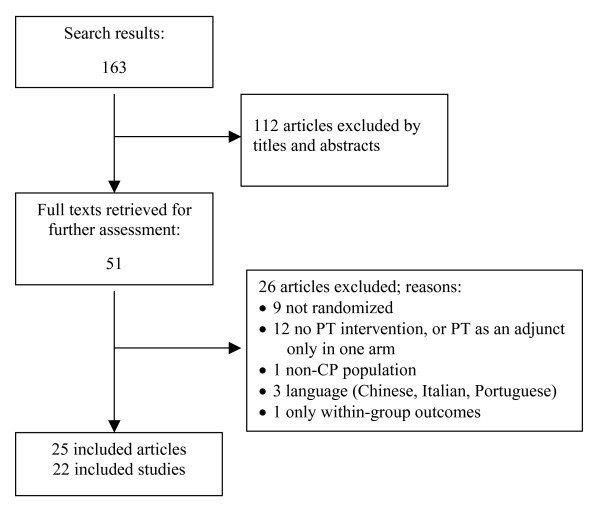
Article selection flow.

The database search identified 163 citations, of which 51 full text articles were retrieved for evaluation (Figure 1). The reasons for exclusion are presented in Additional file 4 (Word document: Articles excluded after reviewing full text and reasons for exclusion). Twenty-five articles describing 22 trials fulfilled all inclusion criteria [34-57]. In three trials the analysis of different outcomes was divided into two reports [39,45,46,48,49,58]. In order to complete the data, one article published before 1990 that had more outcomes than reported in a related paper in 1990 [38] from the same trial was included in the analysis [58]. In one trial we analyzed the data only for the first period which presented a randomized intervention contrast [51]. One trial [39] had four groups (botulinum toxin type A (BTX-A) plus OT, BTX-A alone, OT alone and no-treatment), of which only the two last groups fulfilled the inclusion criteria and were therefore included).

### Methodological quality

The methodological quality scores of the studies are shown in Additional file 5 (Word document: Methodological quality of the trials). Twelve percent of the evaluations, mostly on prognostic similarity and adherence, were resolved by a third reviewer. No trial could blind participants or therapists, and all trials succeeded in similar outcome assessment timing. Four trials fulfilled four important criteria: adequate randomization method, allocation concealment, prognostic similarity and acceptable drop-out rate, and were considered to be high-quality trials [38,40,43,53]. Four other studies fulfilled seven or eight of the quality criteria, but failed to report the randomization method [35], concealment of allocation [36], or whether the groups were different at baseline [37,48].

### Populations and interventions

The characteristics of patients and interventions are summarized in Additional file 6 (Word document: patient and intervention characteristics). All trials were small, recruiting from 10 to 100 children. All types of cerebral palsy were represented. These were classified into various diagnostic subgroups that are somewhat overlapping: spastic diplegia (n = 255), hemiplegia (n = 238), tetraplegia (n = 180), bilateral (n = 56), ataxic or mixed di- or quadriplegia (n = 20), and triplegia (n = 7). The type of spastic CP was not reported in three studies for 52 children [50,56,57]. The age ranged from 7 months to 18 years. In seven trials the children's motor deficit was defined using the Gross Motor Function Classification System and the distribution of the motor impairment was reported as follows: 21% of level I, 20% of level II, 33% of level III, 21% of level IV and 5% of level V [34,35,37,44-46,48,49,55]. In 10 trials the children had mostly mild (51%) or moderate (39%) impairment. Five trials did not report the severity of motor impairment [41-43,54,56]. In three trials some participants were reported to have cognitive impairments [40,51,53].

In one trial all children had undergone multilevel surgery on lower extremities [45,46] and the PT intervention was designed as a postoperative treatment, and in one trial 18 children had had surgery and three botulinum toxin treatments 12 months prior to participation in the trial [48,49]. Stratification techniques were used in twelve trials[34-39,41,42,44,51,55,57,58], usually by age and severity or type of CP and also by gender. One trial stratified the children by the Bayley Scales of Infant Development Mental Developmental Index [39,58] and one by activity and mental function [51].

We formed eight intervention categories. Six trials were classified to comprehensive PT approaches [34-39,58], four to upper extremity treatments, [40-43] four to strength training [44-49], two to cardiovascular fitness or aerobic programs [50,51], two to constraint-induced (CI) therapy [52,53], one to sensorimotor training [54], one to balance training [55], and two to therapy with animals [56,57]. The studied interventions lasted from eight minutes to 12 months (most typically six months). Nine trials had a post-intervention follow-up period (range from one to 18 months from baseline). Full intervention descriptions provided by the trial reports are in Additional file 7 (Word document: Detailed intervention descriptions).

All four strength training trials [44-49] had a no-training comparison group. Seven other trials had a no-extra-therapy comparison group (one trial on upper extremity treatments [40], two cardiovascular fitness and aerobic trials [50,51], one on CI therapy [52], one on balance training [55], and two on therapy with animals[56,57]. Except in the 8-minute trial [56], the children in all groups continued their usual PT [40,43-46,48,49,51,57], or customary care [52], or the add-on therapies were not reported [47,50,55].

Six trials compared two types of interventions [34,36,39,43,53,54,58] and in six trials there was a comparison of another intensity of the same intervention [35,37,38,41-43]. Seven of these 11 trials also included additional therapies for both groups, whereas four trials did not report on this issue [35,36,38,41].

### Effectiveness of interventions

According to the levels of evidence (Table 1) we found no strong evidence on the reviewed interventions, but did establish moderate, limited and conflicting evidence on some particular outcomes in a few intervention categories. The evidence synthesis of the available moderate and conflicting evidence is summarized in Table 2. Moderate evidence was established on the effectiveness of upper extremity treatments and CIMT, and both moderate and conflicting evidence on the strength training depending on the outcomes used. The other intervention categories provided only limited evidence (only one study per intervention).

**Table 2:** The evidence synthesis.

**Study**	**Intervention vs. control**	**Outcome measure**	**Difference between the groups**
**Moderate evidence on effectiveness**			

*Upper extremity treatment (1 high-quality trial)*			
Wallen et al [40]	OT vs. no treatment	Goal Attainment Scale Range of motion in active supination	6 mo: p = 0.054 6 mo: p = 0.008
Hallam [43]	Prehensile hand treatment+NDT vs. NDT (twice a week) vs. NDT (once a week)	GMDS developmental quotient	p < 0.002*
*Constraint induced (CI) therapy (2 lower-quality trials)*			
Charles et al [52]	CI therapy with a sling vs. no therapy	Amount of hand use† Quality of hand use†	1 wk: effect size 0.3, p < .01 1, 6 mo: effect size 0.2, p < 0.01
Taub et al [53]	CI therapy with a cast vs. early intervention program	Amount of hand use‡ Quality of hand use‡	3 wk: p < 0.0001 3 wk: p < 0.0001

**Moderate evidence on ineffectiveness**			

*Strength training (4 lower-quality trials with walking speed, and 2 lower-quality trials with stride length as an outcome)*			
Liao et al [44]	Home-based loaded sit-to-stand exercise vs. no training	Self-selected walking speed	6 wk: NS
Dodd et al [48]	Home-based strength training vs. no training	Self-selected walking speed	6, 18 wk: NS
Patikas et al [45]	Strength training vs. no training	Walking speed Stride length	9 mo: NS 9 mo: NS
Unger et al [47]	Circuit training vs. no training	Walking speed Stride length	9 wk: NS 9 wk: NS

**Conflicting evidence**			

*Strength training (3 lower-quality trials with GMFM as an outcome)*			
Liao et al [44]	Home-based loaded sit-to-stand exercise vs. no training	GMFM	6 wk: effect size 1.17, p = 0.02
Patikas et al [46]	Home-based strength training vs. no training	GMFM	12 mo: NS
Dodd et al [48]	Home-based strength training vs. no training	GMFM	6, 18 wk: NS

mo = months, wk = weeks, GMDF = Griffith's Mental Developmental Scales, GMFM = Gross Motor Function Measure,

* For the prehensile hand treatment+NDT and extra NDT groups compared to NDT group,

† Caregiver Functional Use Survey (14 items, 6-point Likert scale),

‡ Paediatric Motor Activity Log (22 items, scale 0–5)

Differences between study groups in the measured outcomes as classified by ICF components are shown in Additional file 8 (Word document: Effectiveness of physical therapy interventions by the ICF components). Fifty-seven different outcome measures or other endpoints were analyzed: 19 for body functions and structures (range of motion measures for any joint were combined), 32 for activities and participation, two for environmental factors, and four for individual factors. Between-group differences were not analyzed for four outcomes: subjective well-being [40], physical ability and sensory integration[54], and handgrip force [43]. Only eight measures were used in more than one trial: Gross Motor Function Measure (GMFM) in 9 trials, Quality of Upper Extremity Skills Test (QUEST) (n = 4), Peabody Developmental Motor Scales Fine Motor (PDMS-FM) (n = 3), Bruininks-Oseretsky Test of Motor Proficiency (n = 2), Canadian Occupational Performance Measure (COPM) (n = 2), Measure of Processes of Care (MPOC) (n = 2), Modified Ashworth Scale (n = 2), and Pediatric Evaluation of Disability Inventory (PEDI) (n = 2). Full details of the baseline values and changes on all measured outcomes of each trial are presented in Additional file 9 (Word document: Full details of the baseline values and changes on all measured outcomes of each trial).

#### Comprehensive PT approaches

One of the six trials was of high quality [38]. Significant differences between groups were observed in four trials [34-36,39,58]. Use of an Adeli suit in addition to intensive NDT increased mechanical efficiency in stair climbing (limited evidence) [34]. A functional therapy group reached better GMFM scores in standing, walking, running and jumping, and in PEDI for functional skills and caregiver assistance scales, than an NDT group (limited evidence) [36]. Infant stimulation followed by NDT resulted in better motor and mental developmental quotients and independent walking than NDT alone, which had better outcomes only in one sub-item on emotional and verbal responsivity of the mother (limited evidence) [39,58]. An intensive NDT group reached better GMFM-66 scores than a less intensive NDT group, while the group scores did not differ using the GMFM-88 (limited evidence) [35]. The other two trials on different intensities and goal-setting had no between-group differences in GMFM or MPOC [37,38].

#### Upper extremity treatments

Two [40,43] of the four trials [40-43] were of high quality. Significant differences between groups were found in three trials on some outcomes. OT increased active hand supination and goals on various activities (leisure, dressing, eating, postural/weight bearing, school/preschool, other self-care, or other) were achieved more than with no treatment (moderate evidence) [40]. Prehensile hand treatment with NDT twice a week improved the children's developmental status on Griffiths Mental Developmental Scales (GMDS) as compared to NDT once a week (moderate evidence) [43]. NDT with cast increased wrist extension and the quality of hand movement as measured by QUEST compared to NDT with no cast (limited evidence) [42]. No between-group differences were observed in the Child Health Questionnaire, COPM, GMDS, chronological and mental age, MPOC, PEDI, PDMS-FM, and QUEST in the trials where these measures were used (limited evidence).

#### Strength training programs

All the four strength training trials [44-49] were of lower quality. The maximum load of the loaded sit-to-stand test, the physiological cost index [44], and muscle strength [48] improved more in the training than in the no-training groups (limited evidence). In one trial the strength training group performed better in gait analysis, particularly in analyses of the sum of ankle, knee and hip angles at mid-stance compared to the controls, though no differences were found in any of these angles analyzed separately (limited evidence) [47]. No between-group differences were seen in self-selected walking speed [44-49] or in stride length [45-47] measured by gait analysis (moderate evidence). One trial [44] found significant differences between the study groups in the only activity measure used (GMFM), while two trials [46,48] did not (conflicting evidence). Environmental factors were not measured.

Personal factors were considered in two trials [47,49]. Circuit training improved the children's body image but not functional competence on a self-perception scale, as compared to the non-training control group in an African school setting (limited evidence) [47]. In a Canadian home-based training program [49] the results on a Self-perception Profile for Children favored the non-training control group. Their scores improved more in scholastic competence and social acceptance, whereas these scores worsened for the children in the training group (limited evidence). No between-group differences were observed in other sub-items (athletic competence, physical appearance, behavioral conduct) or global self-worth on the same measure (limited evidence).

#### Cardiovascular fitness and aerobic programs

Two lower-quality trials [50,51] measured only outcomes on body functions or structures. An eight-month weight-bearing physical activity program had a positive effect on bone mineral density (limited evidence)[50]. Nine-months of physical training four times per week on top of the normal school sport activities and therapy program had a positive effect on peak aerobic power and improved weight control as compared to a control group (limited evidence) [51]. No effects on physical activity or anaerobic power were observed during the nine-month period (limited evidence).

#### Constraint induced therapy

One high- [53] and one lower-quality [52] trial measured both body functions and structures, and activity and participation outcomes. CI therapy with a cast showed positive effects in the frequency and quality of functional hand use and new emerging behavior as compared to the no-therapy group, but no effects were found on QUEST [53]. CI therapy with a sling had positive effects on functional hand use, time to complete tasks, and speed and dexterity, but no effects on sensibility, handgrip force, or spasticity [52]. Thus there is moderate evidence for the effectiveness of CI therapy on functional hand use.

#### Sensorimotor training programs

One lower-quality trial measured only body functions [54]. The between-group differences were not analyzed, but group treatment had positive short-time within-group effects on sensory integration and physical ability compared to individual therapy (limited evidence).

#### Balance training

One lower-quality trial [55] analyzed dynamic and quiet stance on a force plate and step length of the spastic and non-spastic legs. After six to seven weeks of balance training the children had positive results in displacement in forward and backward direction in quiet stance, in leaning to all directions in dynamic stance, and in the non-paretic leg step length (limited evidence).

#### Therapy with animals

Two lower-quality trials [56,57] on saddle riding on a horse found no between-group differences in muscle symmetry [56] or in any of the seven different outcome measures, except on a sub-item of grasping [57] (limited evidence).

## Discussion

This review did not aim at finding every existing RCT. We started the search from 1990 and searched only databases that most likely would include the relevant papers. We may thus have missed articles if attainable only through e.g. Embase. We did extend our search beyond papers in English, but because of our limited language skills we were not able to judge whether three studies would have fulfilled our inclusion criteria. All included trials were written in English. Relevant studies with inconclusive or negative results may remain unpublished, creating a publication bias.

This systematic review analyzed 22 RCTs on PT interventions in children with CP. All articles except one were published after 1990. Six of these [39,41,42,53,56-58] have been analyzed in previous reviews. Eight different intervention categories were distinguished. The population, interventions and outcomes differed in all categories, which limits comparisons in the evidence synthesis.

The evidence of the effectiveness was considered moderate when it was based on at least one high-quality study or consistent findings in several lower-quality trials (Table 1). Moderate evidence for the effectiveness of two intervention categories on some functional outcomes was established. First, two trials contributed to moderate evidence on upper extremity interventions. In one trial OT resulted in better active supination and individualized goals achieved for various activities compared to no treatment [40] This finding, based on a single trial, is similar to Butler et al's [8] findings that NDT immediately improved dynamic ROM. In another trial, prehensile hand treatment with NDT or NDT provided twice a week improved the children's developmental status as compared to NDT once a week [43] Secondly, constraint-induced therapy resulted in better functional use of the spastic upper extremity compared to conventional therapy [52,53]. Similar conclusions were made in a recent Cochrane review [22].

Furthermore, there was moderate evidence that strength training had no effects on self-selected walking speed based on four trials [44,45,47,48] or on stride length compared to no training based on two trials [45,47]. Conflicting evidence was found on the effectiveness of strength training on gross motor function measured by GMFM compared to no training [44,46,48]. In a previous review [10] effects on walking speed and gross motor function analyzed on the basis of a few observational studies were contradictory and positive, respectively.

There was limited evidence for the other outcomes measured in the upper extremity treatments, strength training and constraint induced therapy trials. For the other five intervention categories (comprehensive PT, cardiovascular fitness and aerobic programs, sensorimotor training, balance training, therapy with animals) there was only one RCT per intervention on the effectiveness of any measured outcome.

Overall, the methodological quality was rather low. Only four trials were of high quality [38,40,43,53]. In most other trials, bias was possible because of a lack of information or deficiencies in the randomization method, group allocation concealment, baseline similarity, number of drop-outs, or in the reporting of co-interventions. Further, some trials did not report on blinding of the outcome assessors or compliance with the intervention. This may of course be just due to poor reporting as described earlier [59]. Further bias may be caused by group differences in the baseline characteristics observed in a third of the trials.

Children with diagnosed CP of all ages between 7 months and 18 years were represented, as well as all CP types and severities. We relied on the authors' description of the diagnosis. None of the included studies reported a significant improvement of motor performance or disappearance of signs indicative of CP, suggesting that the diagnosis of CP had been correct. In some trials the heterogeneity was successfully addressed by stratification. The heterogeneity is a major challenge not only in research, but also when trying to apply the results to children with CP in clinical practice. A toddler with hemiplegia has entirely different goals than an older non-ambulant child with a specific learning disorder. It is important to carefully scrutinize the inclusion criteria for the various studies before clinical application of the evidence.

There were no two similar interventions. Most studies described the interventions well as reported earlier [30,59]. The detailed intervention descriptions allowed for the identification of the active components in each study thus helping to categorize them. The co-interventions, however, remained rather unclear for most of the trials. Children in many trials continued in their usual therapy, the content and intensity of which was not described. These add-on therapies may thus have confounded the outcomes. Even environmental factors, such as parental support, home and leisure time activities, may have an effect on children's functional abilities. These should be recorded and reported similarly for all intervention groups to ensure the possibility of evaluating bias.

The outcome measures varied greatly across the trials. Only eight of the 53 different outcome measures were used in more than one trial. Many of the used measures have not been shown to be sensitive in detecting functional change over time in children with CP [60], except the GMFM and the PEDI [61,62]. International standards are needed to define a core set of outcome measures for follow-up studies in PT interventions. From the viewpoint of ICF most outcome measures were focused on measuring various body functions and structures, and motor activities. The degree of included participation items in the activity measures vary, so one cannot generalize the results to cover also participation. Only few trials measured contextual factors or quality of life. We suggest that environmental factors and the children's overall subjective well-being could also be measured.

We based the evidence synthesis on trial quality and statistical differences in the between-group comparisons in each intervention category. In most studies, however, the differences were reported only using p values, which do not show the effect size. In order to draw clinical conclusions one must rely on the reported baseline and endpoint values for the groups (see Additional file 9, Word document: Full details of the baseline values and changes on all measured outcomes of each trial). Only three trials [35,44,52] presented effect sizes. Small sample sizes in many trials also meant a possibility for type II error i.e. that real group differences could not be detected. A further limitation is that intervention lengths and the timing of measurements varied. Thus caution is necessary when interpreting the results. New trials may change the strength and direction of the evidence. The clinical implications on what interventions to use or not to use in children with CP remain mostly inconclusive.

Comprehensive treatment approaches may be difficult to evaluate in RCT designs for two main reasons. First, the active components of the intervention may vary notably between individuals. Secondly, as the goal of comprehensive intervention is not targeted at specific functions but more on activity or participation, it is more difficult to control confounders, since performance on these levels is affected also by hobbies or other activities at kindergarten, school, or home [30]. A randomized design can more easily be used to evaluate more narrowly defined interventions, such as strength, aerobic, or balance training, or riding.

## Conclusion

This systematic review on trials on children with CP provides some moderate, but mostly limited evidence on the effectiveness of the various PT interventions. Despite the categorization, no exactly similar intervention was studied in more than one trial, so clinical inferences can only be drawn from single studies. Well-designed, randomized trials on current and focused PT interventions are needed, as are new methods for analyzing the effects of comprehensive PT interventions.

## Competing interests

The authors declare that they have no competing interests.

## Authors' contributions

All authors designed the study protocol. HA and JS extracted the data and assessed the study quality of 17 trials, and HA and IA–R of five trials. AM and IA–R provided methodological advice. IA–R interpreted the results into a clinical context. HA analyzed the results and wrote the manuscript. JS, AM, IA–R and MM provided critical revisions to it. All authors read and approved the final manuscript. HA is the guarantor of this article.

## Pre-publication history

The pre-publication history for this paper can be accessed here:



## Supplementary Material

Additional file 1 Search strategy for Ovid Medline. Click here for file

Additional file 2 Quality assessment criteria list and decision rules (adapted and modified from van Tulder et al[33]) Click here for file

Additional file 3 Data extraction form. Click here for file

Additional file 4 Articles excluded after reviewing full text and reasons for exclusion. Click here for file

Additional file 5 Methodological quality of the trials. The four items in italics were considered to constitute "high quality". Click here for file

Additional file 6 Patient and intervention characteristics. Click here for file

Additional file 7 Detailed intervention descriptions. Click here for file

Additional file 8 Effectiveness of physical therapy interventions by the ICF components. Outcomes with moderate evidence are in bold. Click here for file

Additional file 9 Full details of the baseline values and changes on all measured outcomes of each trial. Click here for file

## References

[B1] Rosenbaum P, Paneth N, Leviton A, Goldstein M, Bax M (2006). A report: the definition and classification of cerebral palsy April 2006. Dev Med Child Neurol.

[B2] Bax M, Goldstein M, Rosenbaum P, Leviton A, Paneth N, Jacobsson B, Damiano DL (2005). Proposed definition and classification of cerebral palsy, April 2005. Dev Med Child Neurol.

[B3] Odding E, Roebroeck M, Stam H (2006). The epidemiology of cerebral palsy: incidence, impairments and risk factors. Disabil Rehabil.

[B4] Scrutton D, Damiano DL, Mayston M (2004). Management of the motor disorders of children with cerebral palsy.

[B5] World Confederation for Physical Therapy Description of Physical Therapy. Declarations of principle and position statements. 14th General Meeting of WCPT.

[B6] The Bobath Approach. The Bobath Centre.

[B7] Brown GT, Burns SA (2001). The efficacy of neurodevelopmental treatments in children: a systematic review. Br J Occup Ther.

[B8] Butler C, Darrah J (2001). AACPDM Evidence report: Effects of neurodevelopmental treatment (NDT) for cerebral palsy. Dev Med Child Neurol.

[B9] Tirosh E, Rabino S (1989). Physiotherapy for children with cerebral palsy. Evidence for its efficacy. Am J Dis Child.

[B10] Dodd KJ, Taylor NF, Damiano DL (2002). A systematic review of the effectiveness of strength training programs for people with cerebral palsy.. Arch Phys Med Rehabil.

[B11] Darrah J, Fan JS, Chen LC, Nunweiler J, Watkins B (1997). Review of the effects of progressive resisted muscle strengthening in children with cerebral palsy: a clinical consensus exercise. Pediatr Phys Ther.

[B12] Darrah J, Watkins B, Chen L, Bonin C Effects of conductive education intervention for children with cerebral palsy. AACPDM Evidence Report.

[B13] Pedersen AV (2000). Conductive education - a critical appraisal. Adv Physiother.

[B14] Ludwig S, Leggett P, Harstall C (2000). Conductive education for children with cerebral palsy.

[B15] French L, Nommensen A (1992). Conductive education evaluated: future directions. Aust Occup Ther J.

[B16] Boyd RN, Morris ME, Graham HK (2001). Management of upper limb dysfunction in children with cerebral palsy: a systematic review. Eur J Neurol.

[B17] Hur JJ (1995). Review of research on therapeutic interventions for children with cerebral palsy. Acta Neurol Scand.

[B18] Parette HPJ, Hendricks MD, Rock SL (1991). Efficacy of therapeutic intervention intensity with infants and young children with cerebral palsy. Infants Young Child.

[B19] Horn EM (1991). Basic motor skills instruction for children with neuromotor delays: a critical review. J Spec Educ.

[B20] Morris C (2002). A review of the efficacy of lower-limb orthoses used for cerebral palsy. Dev Med Child Neurol.

[B21] Teplicky R, Law M, Russell D (2002). The effectiveness of casts, orthoses, and splints for children with neurological disorders. Infants Young Child.

[B22] Hoare BJ, Wasiak J, Imms C, Carey L (2007). Constraint-induced movement therapy in the treatment of the upper limb in children with hemiplegic cerebral palsy. Cochrane Database Syst Rev.

[B23] Harris SR, Roxborough L (2005). Efficacy and effectiveness of physical therapy in enhancing postural control in children with cerebral palsy. Neural Plast.

[B24] Pin T, Dyke P, Chan M (2006). The effectiveness of passive stretching in children with cerebral palsy. Dev Med Child Neurol.

[B25] Getz M, Hutzler Y, Vermeer A (2006). Effects of aquatic interventions in children with neuromotor impairments: a systematic review of the literature. Clin Rehabil.

[B26] Snider L, Korner-Bitensky N, Kammann C, Warner S, Saleh M (2007). Horseback riding as therapy for children with cerebral palsy: is there evidence of its effectiveness?. Phys Occup Ther Pediatr.

[B27] Sterba JA (2007). Does horseback riding therapy or therapist-directed hippotherapy rehabilitate children with cerebral palsy?. Dev Med Child Neurol.

[B28] Autti-Rämö I, Suoranta J, Anttila H, Malmivaara A, Mäkelä M (2006). An Overview of Review Articles on the Effectiveness of Upper and Lower Limb Casting and Orthoses Used in Children with Cerebral Palsy. Am J Phys Med Rehabil.

[B29] Siebes RC, Wijnroks L, Vermeer A (2002). Qualitative analysis of therapeutic motor intervention programmes for children with cerebral palsy: an update. Dev Med Child Neurol.

[B30] Kunz R, Autti-Rämö I, Anttila H, Malmivaara A, Mäkelä M (2006). A systematic review finds that methodological quality is better than its reputation but can be improved in physiotherapy trials in childhood cerebral palsy. J Clin Epidemiol.

[B31] Physiotherapy Evidence Database PEDro. http://www.pedro.fhs.usyd.edu.au/index.html.

[B32] World Health Organisation (2001). ICF International classification of functioning, disability and health..

[B33] van Tulder M, Furlan A, Bombardier C, Bouter LM, the Editorial Board of the Cochrane Collaboration Back Review Group (2003). Updated method guidelines for systematic reviews in the Cochrane Collaboration Back Review Group. Spine.

[B34] Bar-Haim S, Harries N, Belokopytov M, Frank A, Copeliovitch L, Kaplanski J, Lahat E (2006). Comparison of efficacy of Adeli suit and neurodevelopmental treatments in children with cerebral palsy. Dev Med Child Neurol.

[B35] Tsorlakis N, Evaggelinou C, Grouios G, Tsorbatzoudis C (2004). Effect of intensive neurodevelopmental treatment in gross motor function of children with cerebral palsy. Dev Med Child Neurol.

[B36] Ketelaar M, Vermeer A, Hart H, van Petegem-van Beek E, Helders PJ (2001). Effects of a functional therapy program on motor abilities of children with cerebral palsy. Phys Ther.

[B37] Bower E, Michell D, Burnett M, Campbell MJ, McLellan DL (2001). Randomized controlled trial of physiotherapy in 56 children with cerebral palsy followed for 18 months. Dev Med Child Neurol.

[B38] Bower E, McLellan DL, Arney J, Campbell MJ (1996). A randomised controlled trial of different intensities of physiotherapy and different goal-setting procedures in 44 children with cerebral palsy. Dev Med Child Neurol.

[B39] Palmer FB, Shapiro BK, Allen MC, Mosher BS, Bilker SA, Harryman SE, Meinert CL, Capute AJ (1990). Infant stimulation curriculum for infants with cerebral palsy: effects on infant temperament, parent-infant interaction, and home environment. Pediatrics.

[B40] Wallen M, O'Flaherty SJ, Waugh MC (2007). Functional outcomes of intramuscular botulinum toxin type a and occupational therapy in the upper limbs of children with cerebral palsy: a randomized controlled trial. Arch Phys Med Rehabil.

[B41] Law M, Russell D, Pollock N, Rosenbaum P, Walter S, King G (1997). A comparison of intensive neurodevelopmental therapy plus casting and a regular occupational therapy program for children with cerebral palsy. Dev Med Child Neurol.

[B42] Law M, Cadman D, Rosenbaum P, Walter S, Russell D, DeMatteo C (1991). Neurodevelopmental therapy and upper-extremity inhibitive casting for children with cerebral palsy. Dev Med Child Neurol.

[B43] Hallam PM (1996). The impact of prehension and fine motor development on gross motor activity in children with cerebral palsy.

[B44] Liao HF, Liu YC, Liu WY, Lin YT (2007). Effectiveness of loaded sit-to-stand resistance exercise for children with mild spastic diplegia: a randomized clinical trial. Arch Phys Med Rehabil.

[B45] Patikas D, Wolf SI, Mund K, Armbrust P, Schuster W, Doderlein L (2006). Effects of a postoperative strength-training program on the walking ability of children with cerebral palsy: a randomized controlled trial. Arch Phys Med Rehabil.

[B46] Patikas D, Wolf SI, Armbrust P, Mund K, Schuster W, Dreher T, Doderlein L (2006). Effects of a postoperative resistive exercise program on the knee extension and flexion torque in children with cerebral palsy: a randomized clinical trial. Arch Phys Med Rehabil.

[B47] Unger M, Faure M, Frieg A (2006). Strength training in adolescent learners with cerebral palsy: a randomized controlled trial. Clin Rehabil.

[B48] Dodd KJ, Taylor NF, Graham HK (2003). A randomized clinical trial of strength training in young people with cerebral palsy. Dev Med Child Neurol.

[B49] Dodd KJ, Taylor NF, Graham HK (2004). Strength training can have unexpected effects on the self-concept of children with cerebral palsy. Pediatr Phys Ther.

[B50] Chad KE, Bailey DA, McKay HA, Zello GA, Snyder RE (1999). The effect of a weight-bearing physical activity program on bone mineral content and estimated volumetric density in children with spastic cerebral palsy. J Pediatr.

[B51] van den Berg-Emons RJ, van Baak MA, Speth L, Saris WH (1998). Physical training of school children with spastic cerebral palsy: effects on daily activity, fat mass and fitness. Int J Rehabil Res.

[B52] Charles JR, Wolf SL, Schneider JA, Gordon AM (2006). Efficacy of a child-friendly form of constraint-induced movement therapy in hemiplegic cerebral palsy: a randomized control trial. Dev Med Child Neurol.

[B53] Taub E, Ramey SL, DeLuca S, Echols K (2004). Efficacy of constraint-induced movement therapy for children with cerebral palsy with asymmetric motor impairment. Pediatrics.

[B54] Bumin G, Kayihan H (2001). Effectiveness of two different sensory-integration programmes for children with spastic diplegic cerebral palsy. Disabil Rehabil.

[B55] Ledebt A, Becher J, Kapper J, Rozendaal RM, Bakker R, Leenders IC, Savelsbergh GJP (2005). Balance training with visual feedback in children with hemiplegic cerebral palsy: effect on stance and gait. Motor Control.

[B56] Benda W, McGibbon NH, Grant KL (2003). Improvements in muscle symmetry in children with cerebral palsy after equine-assisted therapy (hippotherapy). J Altern Complement Med.

[B57] MacKinnon J, Noh S, Lariviere J, MacPhail A, Allan DE, Laliberte D (1995). A study of therapeutic effects of horseback riding for children with cerebral palsy. Phys Occup Ther Pediatr.

[B58] Palmer FB, Shapiro BK, Wachtel RC, Allen MC, Hiller JE, Harryman SE, Mosher BS, Meinert CL, Capute AJ (1988). The effects of physical therapy on cerebral palsy. A controlled trial in infants with spastic diplegia. NEJM.

[B59] Anttila H, Malmivaara A, Kunz R, Autti-Rämö I, Mäkelä M (2006). Quality of reporting of randomized, controlled trials in cerebral palsy. Pediatrics.

[B60] Kirshner B, Guyatt GH (1985). A methodological framework for assessing health indices. J Chronic Dis.

[B61] Vos-Vromans DC, Ketelaar M, Gorter JW (2005). Responsiveness of evaluative measures for children with cerebral palsy: the Gross Motor Function Measure and the Pediatric Evaluation of Disability Inventory.. Disabil Rehabil.

[B62] Ketelaar M, Vermeer A, Helders PJM (1998). Functional motor abilities of children with cerebral palsy: a systematic literature review of assessment measures. Clin Rehabil.

