# Supplementary material for: Effectiveness of physical therapy interventions for children with cerebral palsy: A systematic review
Source: BMC Pediatr. 2008 Apr 24;8:14. doi: 10.1186/1471-2431-8-14 (PMC2390545; doi:10.1186/1471-2431-8-14)
Supplement: Additional file 1 — Search strategy for Ovid Medline. [file 1471-2431-8-14-S1.doc]

**Additional file 1**

Search strategy for Ovid Medline

1. cerebral palsy.mp. or Cerebral Palsy/
9. cerebral palsy/rh, th [Rehabilitation, Therapy]
2. exp physical therapy techniques/
3. (physical therapy or physical therapies).ab,ti.
4. physiotherap$.ab,ti.
5. exp exercise therapy/
6. (physical activity or physical activities).ab,ti.
7. exp "physical therapy (specialty)"/
8. exp "physical education and training"/

10. rehabilitation.mp. or REHABILITATION/
11. (vojta or bobath or neurodevelop$ or NDT or Rood or Kabat or vibroacoust$).ab,ti.
12. "Early intervention (education)"/
13. conductive education.ab,ti.
14. (conservative therap$ or hippo$).mp. or hors$.ab,ti. [mp=title, original title, abstract, name of substance, mesh subject heading]
15. (muscle strength$ or muscle training or motion or therapeutic exercise or exercise training or physical exercise or fitness or aerobic training or kinetic chain training).ab,ti.
16. movement.mp. or EXERCISE MOVEMENT TECHNIQUES/ or MOVEMENT/
17. SWIMMING/ or swimming.mp. or hydrotherapy.mp. [mp=title, original title, abstract, name of substance, mesh subject heading]
18. (functional adj therap$).mp. [mp=title, original title, abstract, name of substance, mesh subject heading]
19. (self adj care adj training).mp. [mp=title, original title, abstract, name of substance, mesh subject heading]
20. occupational therapy.mp. or Occupational Therapy/
21. (constraint adj induced).mp. [mp=title, original title, abstract, name of substance, mesh subject heading]
22. restraint, physical/
23. (forced adj2 treatment).mp. [mp=title, original title, abstract, name of substance, mesh subject heading]
24. (psychomotor performance or sensation).mp. [mp=title, original title, abstract, name of substance, mesh subject heading]
25. sensory integration.mp. or sensory-integration.ab,ti. [mp=title, original title, abstract, name of substance, mesh subject heading]
26. (sensory adj perceptual).mp. [mp=title, original title, abstract, name of substance, mesh subject heading]
27. Parent-Child Relations/ or Parents/ or parent education.mp.
28. physical stimulation.mp. or Physical Stimulation/ or infant stimulation.mp. or Infant Stimulation/
29. exp facilitation/
C) Study design: RCT
30. exp Randomized Controlled Trials/
31. randomized controlled trial.pt.
32. (random$ or rct?).mp. [mp=title, original title, abstract, name of substance, mesh subject heading]
33. 30 or 31 or 32
34. 2 or 3 or 4 or 5 or 6 or 7 or 8 or 11 or 12 or 13 or 14 or 15 or 16 or 17 or 18 or 19 or 20 or 21 or 22 or 23 or 24 or 25 or 26 or 27 or 28 or 29
35. 1 and 33 and 34
36. 1 and 10
37. 9 or 36
38. 33 and 37
