# Supplementary material for: Effectiveness of physical therapy interventions for children with cerebral palsy: A systematic review
Source: BMC Pediatr. 2008 Apr 24;8:14. doi: 10.1186/1471-2431-8-14 (PMC2390545; doi:10.1186/1471-2431-8-14)
Supplement: Additional file 2 — Quality assessment criteria list and decision rules (adapted and modified from van Tulder et al[33]) [file 1471-2431-8-14-S2.doc]

**Additional file 2**

**Quality assessment criteria and decision rules**

Adapted and modified from van Tulder et al [33].

**Criteria list**

1. Was the method of randomization adequate?
2. Was the treatment allocation concealed?
3. Were the groups similar at baseline regarding the most important prognostic indicators?
4. Was the patient blinded to the intervention?
5. Was the care provider blinded to the intervention?
6. Was the outcome assessor blinded to the intervention?
7. Were co-interventions avoided or similar?
8. Was the compliance acceptable in all groups?
9. Was the drop-out rate described and acceptable?
10. Was the timing of the outcome assessment in all groups similar?
11. Did the analysis include an intention-to-treat analysis?

**Decision rules**

A A random (unpredictable) assignment sequence. Examples of adequate methods are computer-generated random number table or similar. Methods of allocation using date of birth, date of admission, hospital numbers, or alternation should not be regarded as appropriate.

B Assignment generated by an independent person not responsible for determining the eligibility of the patients. This person has no information about the persons included in the trial and has no influence on the assignment sequence or on the decision about eligibility of the patient.

C In order to receive a “yes,” groups have to be similar at baseline regarding demographic factors (age, setting), type and severity of CP, types of co-morbidities, and value of main outcome measure(s).

D-F The reviewer determines if enough information about the blinding is given in order to score a “yes.”

G Co-interventions should either be avoided in the trial design or similar between the index and control groups.

H The reviewer determines if the compliance to the interventions is acceptable, based on the reported intensity, duration, number and frequency of sessions for both the index intervention and control intervention(s).

I No dropouts; or the number of participants who were included in the study but did not complete the observation period or were not included in the analysis must be described and reasons given. If the percentage of withdrawals and drop-outs does not exceed 20% for short-term follow-up and 30% for long-term follow-up and does not lead to substantial bias a “yes” is scored.

J Timing of outcome assessment should be identical for all intervention groups and for all important outcome assessments.

K All randomized patients are reported/analyzed in the group they were allocated to by randomization for the most important moments of effect measurement (minus missing values) irrespective of noncompliance and co-interventions.
