# Supplementary material for: Effectiveness of physical therapy interventions for children with cerebral palsy: A systematic review
Source: BMC Pediatr. 2008 Apr 24;8:14. doi: 10.1186/1471-2431-8-14 (PMC2390545; doi:10.1186/1471-2431-8-14)
Supplement: Additional file 3 — Data extraction form. [file 1471-2431-8-14-S3.doc]

# Additional file 3

**Data extraction form**

# Identification

- 1. Evaluation date: ____________________
  2. Reviewer: ____________________
  3. Endnote number: ____________________
  4. Primary author, year: _________________________________________________

# Verification of study eligibility

- Randomized controlled trial
- Children with CP aged 3months - 20 years* at the start of the programme. (* ≥80% of the study population and the data has to be separable.)
- Evaluates physical therapy interventions
- Outcome assessed: functioning

# Study Population

**3.1 Severity and type**

| **Severity scale** | **Hemiplegia** | | | | | **Diplegia** | | | | | **Tetraplegia** | | | | |
| --- | --- | --- | --- | --- | --- | --- | --- | --- | --- | --- | --- | --- | --- | --- | --- |
| What scale? ____________________________________________________________ | Spastic | Dystonic | Ataxic | Mixed | Not specified | Spastic | Dystonic | Ataxic | Mixed | Not specified | Spastic | Dystonic | Ataxic | Mixed | Not specified |
| Levels (as described in the used severity scale) | | | | | | | | | | | | | | | |
|  |  |  |  |  |  |  |  |  |  |  |  |  |  |  |  |
|  |  |  |  |  |  |  |  |  |  |  |  |  |  |  |  |
|  |  |  |  |  |  |  |  |  |  |  |  |  |  |  |  |
|  |  |  |  |  |  |  |  |  |  |  |  |  |  |  |  |
|  |  |  |  |  |  |  |  |  |  |  |  |  |  |  |  |

3.2 Gender and Age

| Group | Gender | | | Age | | | |
| --- | --- | --- | --- | --- | --- | --- | --- |
|  | **Male** | **Female** | **Both** | **Mean** | **SD** | **Median** | **Range** |
| Exercise  ( ) |  |  |  |  |  |  |  |
| Control 1  ( ) |  |  |  |  |  |  |  |
| Control 2  ( ) |  |  |  |  |  |  |  |
| Control 3  ( ) |  |  |  |  |  |  |  |
| **SUM**  (all groups) |  |  |  |  |  |  |  |

**3.3 Comorbidities**

- No
- Yes, list: ____________________________________________________________________
- Not specified

# STUDY INFORMATION

**4.1 Inclusion and exclusion criteria** (page:_________)

- Inclusion criteria: ____________________________________________________________________
- Exclusion criteria: ___________________________________________________________________

**4.2 Stratification**

- No
- Yes, according to what? _______________________________________________________________

**4.3 Flow chart (? = not reported)**

**Assessed for eligibility n=_____**

**Excluded: n=_____**

Not meeting the inclusion criteria (n=___ )

Refused to participate (n= ____ )

Other reasons (n=____ )

**Randomised n= _____**

Lost to follow-up

(n=___ )

Discontinued intervention (n=___ )

**Analysed n=_____**

Excluded from analysis (n=____ )

**Allocated to intervention n=_____**

Received it (n=____ )

Did not receive it (n=___ )

**Enrollment**

**Allocation**

**Intervention follow-up time:_______**

**Analysis after intervention time:_______**

**Allocated to intervention n=_____**

Received it (n=____ )

Did not receive it (n=___ )

Lost to follow-up

(n=___ )

Discontinued intervention (n=___ )

**Analysed n=_____**

Excluded from analysis (n=____ )

**Allocated to intervention n=_____**

Received it (n=____ )

Did not receive it (n=___ )

Lost to follow-up

(n=___ )

Discontinued intervention (n=___ )

**Analysed n=_____**

Excluded from analysis (n=____ )

**Analysed n=_____**

Lost to follow-up

(n=____ )

**Analysed n=_____**

Lost to follow-up

(n=____ )

**Analysed n=_____**

Lost to follow-up

(n=____ )

**Analysed n=_____**

Lost to follow-up

(n=____ )

**Analysed n=_____**

Lost to follow-up

(n=____ )

**Analysed n=_____**

Lost to follow-up

(n=____ )

**Short term follow-up time:_______**

**Long term follow-up time:______**

**Reason(s) for lost to follow-up:** ____________________________________________________________________________

# INTERVENTIONS

**5.1 Index intervention**

**5.1.1 The type of intervention according to authors’ definition** (Description in page: _____)

______________________________________________________________________________

- Neurological intervention
- Other physical therapy intervention
- Therapy with animals

**5.1.2 Dose** (intended and actual length and number of sessions, duration of intervention)

|  | Length of session | Length of session | or hours/week | Number of sessions | Intervention period |
| --- | --- | --- | --- | --- | --- |
| PRE |  |  |  |  |  |
| POST |  |  |  |  |  |

**5.1.3 Setting**

Specify**: ___________________________________________________________________________**

**5.1.4 Delivery type**

- Parent or self-led (e.g. home exercise program)
- Professionally led (1:1)
- Group participation
- Not specified

**5.1.5 Addition of other intervention(s) to index**

- Yes LIST: _____________________________ DOSE:__________________
- No

# Not specified

**5.2 Control group 1**

**5.2.1 The type of intervention according to authors’ definition** (Description in page: _____) ______________________________________________________________________________

- Neurological intervention
- Other physical therapy intervention
- Therapy with animals

**5.2.2 Dose *(intended and actual length and number of sessions, duration of intervention)***

|  | Length of session | Length of session | or hours/week | Number of sessions | Intervention period |
| --- | --- | --- | --- | --- | --- |
| PRE |  |  |  |  |  |
| POST |  |  |  |  |  |

**5.2.3 Setting**

Specify: _________________________________________________________________________

**5.2.4 Delivery type**

- Parent or self-led (e.g. home exercise program)
- Professionally led (1:1)
- Group participation
- Not specified

**5.2.5 Addition of other intervention(s) to comparison intervention**

- Yes LIST: _________________________________________________________________

DOSE:___________________________________________________________________

- No

# Not specified

**5.3 Control Group 2**

**5.3.1 The type of intervention according to authors’ definition** ______________________________________________________________________________

- Neurological intervention
- Other physical therapy intervention
- Therapy with animals

**5.3.2 Dose *(intended and actual length and number of sessions, duration of intervention)***

|  | Length of session | Length of session | or hours/week | Number of sessions | Intervention period |
| --- | --- | --- | --- | --- | --- |
| PRE |  |  |  |  |  |
| POST |  |  |  |  |  |

**5.3.3 Setting**

Specify: _________________________________________________________________________

**5.3.4 Delivery type**

- Parent or self-led (e.g. home exercise program)
- Professionally led (1:1)
- Group participation
- Not specified

**5.3.5 Addition of other intervention(s) to comparison intervention**

- Yes LIST: _____________________________________________________________

DOSE:________________________________________________________________

- No

# Not specified

**5.4 Comparisons**

- Sham or ineffective treatments
- Effective treatments
- No treatment

# Outcome

**6.1 Outcome measure(s) assessed**

- Body function & structures
- Activity limitation & participation restriction

**6.2 Measures and timing (underline the primary)**

| **Measures** | **Before baseline** | **Baseline** | **At the end** | **Short term** | **Long term (<6m)** |
| --- | --- | --- | --- | --- | --- |
|  |  |  |  |  |  |
|  |  |  |  |  |  |
|  |  |  |  |  |  |
|  |  |  |  |  |  |
|  |  |  |  |  |  |
|  |  |  |  |  |  |
|  |  |  |  |  |  |
|  |  |  |  |  |  |
|  |  |  |  |  |  |

**6.3 Outcome assessments**

Instrument _________________________________________________

Scale:______________________

Clinical significance level:_______________________________________________

| After THE intervention ______ weeks/months | **Physical therapy** | **Control 1**  **( )** | **Control 2**  **( )** | **Control 3**  **( )** |
| --- | --- | --- | --- | --- |
| Total number of patients |  |  |  |  |
| Number improved* |  |  |  |  |
| Percentage improved |  |  |  |  |
| Baseline mean (SD)  (Range) |  |  |  |  |
| Post-mean (SD)  (Range) |  |  |  |  |
| Mean change (SD)  (Range) |  |  |  |  |
| (95% CI) of mean change |  |  |  |  |
| Statistical test: |  |  |  |  |
| p-value |  |  |  |  |

* How improvement was described / defined? _______________________________________

| follow-up ______ wk/mo | **Physical therapy** | **Control 1**  **( )** | **Control 2**  **( )** | **Control 3**  **( )** |
| --- | --- | --- | --- | --- |
| Total number of patients |  |  |  |  |
| Number improved* |  |  |  |  |
| Percentage improved |  |  |  |  |
| Baseline mean (SD)  (Range) |  |  |  |  |
| Follow-up mean (SD)  (Range) |  |  |  |  |
| Mean change (SD)  (Range) |  |  |  |  |
| (95% CI) of mean change |  |  |  |  |
| Statistical test: |  |  |  |  |
| p-value |  |  |  |  |

* How improvement was described / defined? _____________

**6.4 Adverse Effects**

- No adverse effects
- Adverse effects

Type: __________________________________________________

Number of index group: ________

Number of control group: ________

- Not specified

# CONCLUSIONS

| **Outcome measure** | **Index outcome** | | | |
| --- | --- | --- | --- | --- |
|  | **Positive** | **Neutral** | **Negative** | **Unclear** |
| Body structure/function |  |  |  |  |
| Activity limitation |  |  |  |  |
| Participation restriction |  |  |  |  |
| Environmental factors |  |  |  |  |
| Personal factors |  |  |  |  |
|  | | | | |
| Authors’ overall |  |  |  |  |
| Reviewer's overall |  |  |  |  |

**NOTES**:________________________________________________
