# Supplementary material for: Effectiveness of physical therapy interventions for children with cerebral palsy: A systematic review
Source: BMC Pediatr. 2008 Apr 24;8:14. doi: 10.1186/1471-2431-8-14 (PMC2390545; doi:10.1186/1471-2431-8-14)
Supplement: Additional file 4 — Articles excluded after reviewing full text and reasons for exclusion. [file 1471-2431-8-14-S4.doc]

**Additional file 4**

Articles excluded after reviewing full text and reasons for exclusion.

| **References (n=26)** | **Reason for exclusion** |
| --- | --- |
| Adams MA, Chandler LS, Schuhmann K. Gait changes in children with cerebral palsy following a neurodevelopmental treatment course. *Pediatr Phys Ther*. 2000;12:114-20 | Not randomized |
| Catanese AA, Coleman GJ, et al. Evaluation of an early childhood programme based on principles of conductive education: the Yooralla project. *J Paediatr Child Health.* 1995;31:418-22 | Not randomized |
| Cherng R, et al. The effectiveness of therapeutic horseback riding in children with spastic cerebral palsy. *Adapt Phys Act Q* 2004; 21:103-21 | Not randomized |
| Fetters L, Kluzik J. The effects of neurodevelopmental treatment versus practice on the reaching of children with spastic cerebral palsy. *Phys Ther* 1996;76: 346-58 | Not randomized |
| [Palisano RJ](http://www.ncbi.nlm.nih.gov/sites/entrez?Db=pubmed&Cmd=Search&Term="Palisano RJ"%5BAuthor%5D&itool=EntrezSystem2.PEntrez.Pubmed.Pubmed_ResultsPanel.Pubmed_RVAbstractPlus), [Tieman BL](http://www.ncbi.nlm.nih.gov/sites/entrez?Db=pubmed&Cmd=Search&Term="Tieman BL"%5BAuthor%5D&itool=EntrezSystem2.PEntrez.Pubmed.Pubmed_ResultsPanel.Pubmed_RVAbstractPlus), et al. Environmental setting on mobility methods of children with cerebral palsy. *Dev Med Child Neurol* 2003; 45:113-20 | Not randomized |
| Ross SA, Engsberg JR, et al. Ankle strengthening to improve gait and function in cerebral palsy - a pilot study. *Pediatr Phys Ther.* 2006;18:80-1 | Not randomized |
| Sung IY, Ryu JS, et al. Efficacy of forced-use therapy in hemiplegic cerebral palsy. *Arch Phys Med Rehabil* 2005;86:2195-8 | Not randomized |
| Thorpe DE, Valvano J. The effects of knowledge of performance and cognitive strategies on motor skill learning in children with cerebral palsy. *Pediatr Phys Ther.* 2002;14:2-15 | Not randomized |
| Tieman BL, Palisano RJ, et al. Changes in mobility of children with cerebral palsy over time and across environmental settings. *Phys Occup Ther Pediatr.* 2004; 24:109-28 | Not randomized |
| Mayo NE. The effect of physical therapy for children with motor delay and cerebral palsy. A randomized clinical trial. *Am J Phys Med Rehabil.* 1991;70:258-67 | Population: over 20 % of participants non-CP. |
| Burditt CA. The effects of therapeutic taping on seated postural control in children with cerebral palsy, quadriplegia. Dissertation. University of Miami, 1999. | Intervention: taping as an adjunct to physiotherapy |
| Coleman GJ, King JA, et al. A pilot evaluation of conductive education-based intervention for children with cerebral palsy: the Tongala project. *J Paediatr Child Health* 1995;31:412-7 | Intervention: conductive education |
| Duff SV, Gordon AM. Learning of grasp control in children with hemiplegic cerebral palsy. *Dev Med Child Neurol.* 2003; 45:746-57 | Intervention: grasp control as an adjunct to physiotherapy |
| Duncan B, Barton L, et al. Parental perceptions of the therapeutic effect from osteopathic manipulation or acupuncture in children with spastic cerebral palsy. *Clin Pediatr.* 2004;43:349-53 | Intervention: osteopathic manipulation vs. acupuncture |
| Dursun, E, Dursun N, Alican D. Effects of biofeedback treatment on gait in children with cerebral palsy. *Disabil Rehabil.* 2004; 26:116-20 | Intervention: biofeedback as an adjunct to physiotherapy |
| Kramer JF, Ashton B, et al. Training of head control in the sitting and semi-prone positions. *Child: Care, Health & Development.* 1992;18:365-76 | Intervention: training of head control semi prone vs. sitting training position |
| McConachie, H, Huq S, et al. A randomized controlled trial of alternative modes of service provision to young children with cerebral palsy in Bangladesh. *J Pediatr.* 2000; 137:769-76 | Intervention: center-based mother-child group vs. monthly training of parents along with a pictorial guidance manual |
| Reddihough, DS, King J, et al. Efficacy of programmes based on conductive education for young children with cerebral palsy. *Dev Med Child Neurol.* 1998; 40:763-70 | Intervention: conductive education vs no treatment. |
| Steinbok P, McLeod K. Comparison of motor outcomes after selective dorsal rhizotomy with and without preoperative intensified physiotherapy in children with spastic diplegic cerebral palsy. *Pediatr Neurosurg.* 2002;36(3):142-7 | Intervention: selective dorsal rhizotomy as an adjunct to physiotherapy |
| Steinbok P, Reiner AM, et al. A randomized clinical trial to compare selective posterior rhizotomy plus physiotherapy with physiotherapy alone in children with spastic diplegic cerebral palsy. *Dev Med Child Neurol.* Mar 1997;39(3):178-84. | Intervention: selective dorsal rhizotomy as an adjunct to physiotherapy |
| Stiller C, Marcoux BC, et al. The effect of conductive education, intensive therapy, and special education services on motor skills in children with cerebral palsy. *Phys Occup Ther Pediatr.* 2003;23:31-50 | Intervention: conductive education, intensive therapy and special education |
| Wallen MA, O'Flaherty SJ, et al. Functional outcomes of intramuscular botulinum toxin type A in the upper limbs of children with cerebral palsy: a phase II trial." *Arch Phys Med Rehabil.* 2004;85:192-200 | Intervention: botulinum toxin A, no PT |
| Liu J. The influence of the early interference in the convalescence on the infantile cerebral palsy. *Modern Rehabil.* 2000;4:844-5 | Language: Chinese |
| Pisaturo C, et al. La paralisi cerebrale ipotonica. Quale trattamento? [Hypotonic cerebral palsy. Which treatment?]. *Minerva Pediatrica.* 1997;49:551-8 | Language: Italian |
| Tudella E, Formiga CKM, et al. Comparison of the effectiveness of the early and late physical therapy intervention in infants with cerebral palsy. *Fisioterapia em Movimento*. 2004;17:45-52 | Language: Portuguese |
| DeLuca SC, Echols K, Law CR, Ramey SL. Intensive pediatric constraint-induced therapy for children with cerebral palsy: randomized, controlled, crossover trial. *J Child Neurol.* 2006;21(11):931-8. | Reports only within-group data of the trial by Taub et al.[53] |
