# Supplementary material for: Effectiveness of physical therapy interventions for children with cerebral palsy: A systematic review
Source: BMC Pediatr. 2008 Apr 24;8:14. doi: 10.1186/1471-2431-8-14 (PMC2390545; doi:10.1186/1471-2431-8-14)
Supplement: Additional file 5 — Methodological quality of the trials. The four items in italics were considered to constitute "high quality". [file 1471-2431-8-14-S5.doc]

**Additional file 5**

**Methodological quality of the trials. The four items in italics were considered to constitute "high quality".**

| **First author (year)** | ***Adequate randomi-zation*** | ***Allo-cation conceal-ment*** | ***Prog-nostic similarity*** | **Subject blinding** | **Therapist blinding** | **Assessor blinding** | **Co interventions avoided or similar** | **Accept-able comp-liance** | ***Accept-able and described drop-out rate*** | **Similar outcome assessment timing** | **Intention to treat analysis** | **No of "yes" scores** |
| --- | --- | --- | --- | --- | --- | --- | --- | --- | --- | --- | --- | --- |
| ***Comprehensive physiotherapy programs*** | | | | | | | | | | | | |
| Bar-Haim (2006) | *yes* | *?* | *yes* | no | no | ? | yes | ? | *yes* | no | yes | 5 |
| Tsorlakis (2004) | *?* | *yes* | *yes* | no | no | yes | ? | yes | *yes* | yes | yes | 7 |
| Ketelaar (2001) | *yes* | *?* | *yes* | no | no | yes | yes | yes | *yes* | yes | yes | 8 |
| Bower (2001) | *yes* | *yes* | *no* | no | no | yes | yes | no | *yes* | yes | yes | 7 |
| Bower (1996) | *yes* | *yes* | *yes* | no | no | yes | ? | *yes* | *yes* | yes | yes | 8 |
| Palmer (1990, 1988) | *?* | *?* | *yes* | no | no | no | ? | yes | *yes* | yes | yes | 5 |
| ***Upper extremity treatment*** | | | | | | | | | | | | |
| Wallen (2007) | *yes* | *yes* | *yes* | no | no | no | ? | *yes* | *yes* | yes | yes | 7 |
| Law (1997) | *?* | *?* | *yes* | no | no | yes | ? | yes | *yes* | yes | yes | 5 |
| Law (1991) | *?* | *?* | *yes* | no | no | yes | ? | yes | *yes* | yes | yes | 6 |
| Hallam (1996) | *yes* | *yes* | *yes* | no | no | ? | yes | *yes* | *yes* | yes | yes | 8 |
| ***Strength training programs*** | | | | | | | | | | | | |
| Liao (2007) | *?* | *?* | *yes* | no | no | yes | ? | yes | *yes* | yes | yes | 6 |
| Patikas (2006) | *yes* | *yes* | *?* | no | no | no^45^, yes ^46^ | yes | ? | *yes* | yes | no | 6 |
| Unger (2005) | *yes* | *?* | *yes* | no | no | yes | ? | ? | *yes* | yes | ? | 5 |
| Dodd (2003, 2004) | *yes* | *yes* | *no* | no | no | yes | yes | yes | *yes* | yes | yes | 8 |
| ***Cardiovascular fitness and aerobic programs*** | | | | | | | | | | | | |
| Chad (1999) | *?* | *?* | *yes* | no | no | ? | ? | ? | *yes* | yes | yes | 4 |
| Van Den Berg-Emons (1998) | *?* | *?* | *yes* | no | no | ? | ? | yes | *yes* | yes | yes | 5 |
| ***Constraint induced therapy*** | | | | | | | | | | | | |
| Charles (2006) | *?* | *?* | *yes* | no | no | yes | ? | yes | *no* | yes | ? | 4 |
| Taub (2004) | *yes* | *yes* | *yes* | no | no | no | ? | *yes* | *yes* | yes | yes | 7 |
| ***Sensorimotor training*** | | | | | | | | | | | | |
| Bumin (2001) | *no* | *no* | *?* | no | no | no | ? | ? | *no* | yes | yes | 2 |
| ***Balance training*** | | | | | | | | | | | | |
| Ledebt (2005) | *?* | *?* | *no* | no | no | no | ? | ? | *no* | yes | ? | 1 |
| ***Therapy with animals*** | | | | | | | | | | | | |
| Benda (2003) | *?* | *yes* | *?* | no | no | no | yes | yes | *yes* | yes | yes | 6 |
| MacKinnon (1995) | *?* | *?* | *?* | no | no | yes | ? | yes | *yes* | yes | yes | 5 |

Yes=criteria fulfilled, no=criteria not fulfilled, ?=don't know.
