# Supplementary material for: Effectiveness of physical therapy interventions for children with cerebral palsy: A systematic review
Source: BMC Pediatr. 2008 Apr 24;8:14. doi: 10.1186/1471-2431-8-14 (PMC2390545; doi:10.1186/1471-2431-8-14)
Supplement: Additional file 6 — Patient and intervention characteristics. [file 1471-2431-8-14-S6.doc]

**Additional file 6 Patient and intervention characteristics. (Table continues)**

|  | **Participants** | | **Interventions** | | | |
| --- | --- | --- | --- | --- | --- | --- |
| **First author, (year)** | **a. N (group n)**  **b. Age-range, mean (±SD)**  **c. Sex distribution** | **d. Type of CP,**  **e. Severity of motor deficit** | **Intervention comparisons** | **Intensity**  ***[realized intensity]*** | **Add-on interventions in all groups** | **Setting and provider** |
| ***Comprehensive physiotherapy programs*** | | | | | | |
| Bar-Haim (2006) | a. 24 (12/12)  b. 5y2mo-12y 11mo, 8y2mo  I: 8.3y (**±**2.0), C: 8.1y (**±**2.2)  c. I: 8 M, 4 F, C: 9 M, 3 F | d. I: 6 spastic/ataxic diplegia, 1 triplegia, 5 spastic/mixed quadriplegia  C: 5 spastic diplegia, 7 spastic/mixed quadriplegia  e. GMFCS  I: 2 II, 6 III, 4 IV;  C: 2 II, 5 III, 5 IV. | I: NDT with Adeli suit  C: NDT | I: 120 min, 5 x wk for 4 wk (20 sessions)  C: 120 min, 5 x wk for 4 wk (20 sessions) | Both groups stopped their routine pt treatments, but continued educational and recreational activities (dose, intensity and number not defined). | I: Russian pts, experts in Adeli suit application, same environment for all children. C: pts with ≥7y experience and training of NDT basic and advanced courses; rehabilitation centre. |
| Tsorlakis (2004) | a. 34 (17/17)  b. 3-14y, 7y 3mo (**±**3y6mo)  c. 14 F, 24 M | d. 10 spastic hemiplegia, 12 diplegia and 12 tetraplegia.  e. GMFCS: 10 I, 10 II, 14 III. | I: Intensive NDT  C: NDT | I: 50min, 5 x wk  C: 50 min, 2 x wk | Not reported | 17 pts with NDT certification for at least 5 years and at least 10 year clinical experience. |
| Ketelaar (2001) | a. 55 (28/27)  b. 24-87mo, 55mo (**±**20)  c. Not reported | d. 32 hemi-, 11 di- and 12 tetraplegia.  e. 43 mild and 12 moderate. | I: Functional PT  C: Continued previous PT regime | I: Mean frequency at baseline: 3.4 x mo) *[45min/session]*  C: Mean frequency at baseline: 3.8 x mo) *[45 min/session]* | Not reported | Pediatric pts working in primary health care, number not given. |
| Bower (2001) | a. 56 (15/13/13/15)  b. 3-12y  c. Not reported | d. Bilateral CP.  e. GMFCS: 17 III, 29 IV, 10 V. | I: Intensive PT + generalized aims  C1: Intensive PT + individual and measurable treatment goals  C2: Routine PT + generalized aims  C3: Routine PT + individual and measurable treatment goals | I: 60min, 5 x wk *[mean 44 h/mo]*  C1: 60 min, 5 x wk *[mean 44 h/mo]*  C2: Routine amount *[mean 6 h/3mo]*  C3: Routine amount *[mean 6 h/3mo]* | Routine amounts of therapy on equipment, orthotics, and on consultation. Large individual variation in the amounts activities e.g. hydrotherapy, horse riding, occupational therapy, school physical education, conductive education. | 56 pts (child's own) at current praxis. |
| Bower (1996) | a. 44 (11/11/11/11)  b. 3-11y  c. Not reported | d. Quadriplegic CP.  e. SRCMD: 28 moderate, 16 severe. | I: Conventional PT + generalized aims  C1: Intensive PT + generalized aims  C2: Conventional PT + individual and measurable treatment goals  C3: Intensive PT + individual and measurable treatment goals | I: Conventional amount *[2,0 (range 1,0-3,0) h/ 2 wk]*  C1: 60min, 5 x wk *[9,2 (range 8,0-10,0) h /2 wk]*  C2: Conventional amount *[2,2 (range 1,0-3,0) h/2 wk]*  C3: 60min, 5 x wk *[9,3 (range 6,0-10,0) h/2 wk]* | Not reported | 44 pts (child's own) at current praxis. |
| Palmer (1990, 1988) | a. 48 (25/23)  b. 12-19mo  c. Not reported | d. Spastic diplegia.  e. Mild to severe. | I: NDT  C: Infant stimulation (6 mo) + NDT (6 mo) | I: Home prg: daily (amount not given); PT 60min in 14 d; *[>90 % compliance]*  C: Home prg: daily (amount not given); PT 60 min in 14 d *[>90 % compliance]* | No additional therapies | Parents at home and therapist at the Clinical Research Unit of the Kennedy Institute for Handicapped Children. |
| ***Upper extremity treatment*** | | | | | | |
| Wallen (2007) | a. 32 (17/15)*  b. 2-11y, I: 5y2mo (±2y11mo); C: 5y11m (±2y10mo)  c. I: 53% M, C: 73% M. | d. I: 8 hemiparesis, 3 triplegia, 6 quadriparesis; (C) 8 hemiparesis, 2 triplegia, 5 quadriparesis.  MAS score of 2: I: 77%, C: 73%.  e. Motor control scale developed for study†: I: I 12%, II 47%, III 35%, IV 6%, C: I 6%, II 33%, III 53%, IV 13%. | I: OT  C: No extra OT. | I: 60min, 1 x wk, for 12 wk.  C: Not reported. | Pre-existing levels of regular therapy was maintained (dose, intensity and number not defined). | The Children's Hospital at Westmead. or the children's usual OTs |
| Law (1997) | a. 50 (26/24)  b. 18mo - 4y, 32.92mo  c. Not reported | d. 19 hemiplegia, 9 diplegia, 22 tetraplegia.  e. Not reported. | I: Intensive NDT + casting + home prg  C: Regular OT | I: NDT: 45min, 2 x wk; Home prg: 30min daily; Casting: minimum 4 h daily  C: 45min, 1-4 x mo | Not reported | 8 different rehabilitation centers in Ontario, Canada; ots, number not given. |
| Law (1991) | a. 72 (19/17/18/18)  b. 18mo - 8y  c. Not reported | d. 44 Spastic hemiplegia, 28 tetraplegia.  e. Not reported. | I: Intensive NDT + cast  C1: Intensive NDT without casts  C2: Regular NDT + casts  C3: Regular NDT without casts | I: NDT: 45 min, 2 x wk *[mean 45min/wk]* Home prg: 30min daily *[on 66% more than 75% of time]*; Casting: ≥4 h/d *[mean 3.05 h/d]*  C1: NDT: 45 min, 2 x wk *[mean 45min/wk]*; Home prg.: 30min daily *[on 66% more than 75% of time]*  C2: NDT: 1-4 x mo *[mean 11 sessions/6 mo]*; Home prg: 15min, 3 x wk *[on 66% more than 75% of time]*;  C3: Casting: ≥ 4 h/d *[ mean 3.05 h/d]*  NDT: 1-4 x mo *[mean 11 sessions/6 mo]*; Home prg: 15min, 3 x wk *[on 66% more than 75% of time]* | Records were kept of other type of interventions during six mo intervention. | 3 regional OT centers; ots, number not given. |
| Hallam (1996) | a. 100 (33/33/34)  b. Not reported  c. 58 M, 42 F | d. 13 diplegia, 18 right hemiplegia, 22 left hemiplegia, 37 quadriplegia, 10 double diplegia (arms affected more than legs).  e. Not reported. | I: Prehensile hand treatment  C1: NDT  C2: No extra NDT or hand therapy | I: 1 x wk *[mean 20 (range 15-29) sessions]*  C1: 1 x wk *[mean 19 (range 12-27) sessions]*  C2: - | Traditional PT (NDT) 1 x wk. | I: the researcher pt, PT was provided by the child's own pt. |
| ***Strength training programs*** | | | | | | |
| Liao (2007) | a. 24 (12/12)  b. 5-12y;  I: 85.6mo (±20.8),  C: 91.3mo (±17.5)  c. 8 F, 12 M | d. Spastic diplegia.  e. GMFCS: I: 4 I, 6 II, C: 6 I, 4 II. | I: Home-based loaded sit-to-stand resisted exercise prg  C: No extra prg | I: 3 sets/session, 3 x wk for 6 wk with increasing loads every 2 wk *[duration 20-30 min, mean loads: 4.8 kg (1-2 w), 5.5 kg (3-4w), 6.3 kg (5-6w)]*  C: - | PT including passive ROM exercises, positioning, balance training, functional training, NDT. I: 1xwk (n=4), 2xwk (n=2), discontinued PT (n=4);  C: 1xwk (n=5), 2xwk (n=1), no PT (n=3). | Home with caregiver assistance. Trainer taught and checked the exercises other every other wk visits at home or laboratory. |
| Patikas (2006) | a. 43 (21/22)  b. 6-16y, 9.7y (±2.8)  c. 12 F, 27 M | d. Spastic diplegia.  e. GMFCS: 12 I, 18 II, 9 III. | I: Strength training + PT  C: No training + PT | I: 30-45 min, 3-4 x wk, for 9 mo *[mean 3.2±0.3 x wk, for 40.3±0.4 wk].*  C: - | Conventional PT after surgery as soon as mobilization was possible. | I: Home, self-led with help of parents. I & C: 4 pts during hospital stay and after that by the children's own pts. |
| Unger (2005) | a. 31 (24/13)  b. 13-18 y; I: 13.5-18.92y (**±**15.86); C: 14-18.33y (**±**16.28)  c. 12 F, 19 M | d. Spastic CP. 16 hemiplegic, 14 diplegic, 1 triplegia.  e. Independently ambulant with or without aids. Assistive devices: I: crutches (n=1), wheelchair in occasional use (n=1), supra-malleolar orthosis (n=1). | I: Circuit training  C: No training. | I: 1-3 x wk for 8 wk during school hours.  C: - | Not reported. | I: School, consultation by therapist, research assistant. |
| Dodd (2003, 2004) | a. 21 (11/10)  b. 8-18y, 13y1mo (**±**3y1mo) c. 10 F/11 M | d. Spastic diplegia.  e. GMFCS: 7 I, 5 II, 9 III. | I: Home-based strength-training prg  C: Normal daily activity | I: 20-30 min, 3 x wk  C: - | Normal PT prg (45min 1-2 x mo), normal daily activities, including school and sport. | I: Home, self-led.  C: school; pt checked exercise performance and adjusted the load at 2 wk intervals. |
| ***Cardiovascular fitness and aerobic programs*** | | | | | | |
| Chad (1999) | a. 18 (9/9)  b. range not given; I: 9.0y (**±**2.9); C: 9.0 y (**±**2.7) c. 13 F, 5M | d. Spastic CP.  e. 2 independent ambulators, 5 independent ambulators with an aid, 5 ambulators with an assistant, 6 non-ambulators. | I: Physical activity prg  C: Maintenance of normal lifestyle habits. | I: 2 x wk for 2 mo, 3 x wk for 6 mo.  C: - | Not reported. | Setting not reported; pt one-on-one, number not given. |
| Van den Berg-Emons (1998) | a. 20 (10/10)  b. 7-13y, 9y (±1.4)  c. 9 F, 11 M | d. 14 spastic diplegia and 2 mixed spastic and ataxic diplegia, 4 tetraplegia.  e. 10 ambulant, 10 wheelchair-bound. | I. Physical training prg  C: No training. | I: 45 min, 4 x wk (+additional therapy) *[84%]*  C: - | School prg: 45 min gymnastic lessons 2xwk; individual therapy prg based on personal needs; frequency varying from no therapy to >2.5 hours/wk. | Not reported |
| ***Constraint induced therapy*** | | | | | | |
| Charles (2006) | a. 33 (19/14)  b. 4-8y, 6y 8mo (**±**1y 4mo)  c. 8 F, 14 M | d. Hemiplegic CP. Involved side I: 8 left, 3 right, C: 4 left, 7 right.  e. Moderate hand involvement ‡, a 50% difference between the involved and non-involved hand on the Jebsen-Taylor Test. | I: CI-therapy with a sling  C: No treatment. | I: 6 h/session for 10 out of 12 consecutive d, altogether 60 h. *[At least 5h 45min/d wearing the sling, structured training 29-50h, mean 35h. In addition home prg using their involved extremity 5.7 h/10 d during the intervention and 7.3 h/wk for 6mo after the intervention.]*  C: - | The children continued to receive the usual and customary care that the children were receiving elsewhere. | I: Columbia university, a trained interventionist involving specific practice of designated target movements. |
| Taub (2004) | a. 18 (9/9)  b. 7mo-8y, 41.5mo  c. 5 F, 13 M | d. Spastic or low muscle tone hemiparesis.  e. 2 mild, 7 moderate, 2 moderate-severe, 7 severe. | I: CI-therapy with a bivalved cast  C: Early intervention prg, OT and/or PT  (+ crossover to CI therapy for 21 d) | I: 6 h/session, 7 x wk (21 d in total)  C: 1-2 h/wk, *[mean 2.2 h/wk, range 1 session/21 d to 4 h/wk]* | Not reported | I: Natural settings, ots and pts, or a pt assistant; 1 method developer trained and supervised the therapists twice wkly.  C: previously established school-based services or private therapy sessions. |
| ***Sensorimotor training*** | | | | | | |
| Bumin (2001) | a. 41 (16/16/9)  b. range not given; I: 7,06y (**±**1,88); C1: 7,68y (**±**1,70); C2: 7,00y (**±**1,22)  c. Not reported | d. Spastic diplegia.  e. Not reported. | I: Sensory perceptual motor (SPM) training individually.  C1: SPM training in groups of 4.  C2: Home prg | I: 90min, 3 x wk  C1: 90min, 3 x wk  C2: Not reported. | Some activities as a home prg. | I, C1: The school of PT and rehabilitation Hacettepe University OT Unit  C2: home; pts, number not given. |
| ***Balance training*** | | | | | | |
| Ledebt (2005) | a. 10 (?/?)  b. 5-10 y; I: 7y2mo, C: 7y7mo  c. Not reported | d. Hemiplegic CP  e. GMFCS: I 10. | I: Balance training  C: No balance training. | I: 30 min, 3 x wk for 6 wk (18 sessions)  C: - | Own shoes including ankle-foot orthoses or insoles to correct leg-length discrepancies. | University laboratory in Amsterdam, 2 trainers. |
| ***Therapy with animals*** | | | | | | |
| Benda (2003) | a. 15 (7/8)  b. 4-12y  c. Not reported | d. Spastic CP.  e. Not reported. | I: Equine-assisted therapy (Hippo therapy)  C: Stationary barrel | I: one 8 min session  C: one 8 min session | No additional therapies. | Therapeutic Riding of Tuscon, 1 pt certified as a hippo therapy clinical specialists. |
| MacKinnon (1995) | a. 19 (10/9)  b. 4-12y, 6.5y ; I: 7.2 (±2.39), C1: 5.7y (±1.46), C2: 6.8y (±2.05), C3: 6.0 (±1.87)  c. 10 F, 9 M | d. Spastic CP.  e. 10 mild, 9 moderate. | I: Horseback riding, moderate  C1: No hippo therapy (waiting list), moderate  C2: Horseback riding, mild  C3: No hippo therapy (waiting list), mild | I: 60 min, 1 x wk *[93.1%]*  C1: -  C2: 60 min, 1 x wk *[93.1%]*  C3: - | Routine therapies and activities continued, no attempt to stop them was made. | Setting not reported; 1 therapeutic riding instructor and 1 pt. |

I=intervention group, C=control group, F=females, M=males, min=minutes, h=hours, d=day/s, wk=week/s, mo=month(s), y=year(s), CP=cerebral palsy, pt=physiotherapist, ot=occupational therapist, prg=program, PT=physiotherapy, OT=occupational therapy,

CI=constraint inducement, NDT=neurodevelopmental treatment, SPM=sensory perceptual motor, MAS=Modified Ashworth scale, DASI-II=Developmental Activities Screening Inventory, SRCMD = Standard Record of Central Motor Deficit: section 7; GMFCS = Gross Motor Function Classification System (levels I-V).

*The Wallen 2007 trial included a total of 80 participants who were randomized to four groups. The 2 other groups (BTX-A plus OT and BTX-A) were not included in this review.

† I=manipulate small objects, pincer, opposition of most fingers, II=useful grasp and release for holding larger objects, III=can flex and extend fingers and wrist, IV=movement that is not useful for activity.

‡ type IIa by Zancolli EA, Zancolli ER: Management of the hemiplegic spastic hand in cerebral palsy. Surg Clin North Am 1981, 61:395-406.
