# Supplementary material for: Effectiveness of physical therapy interventions for children with cerebral palsy: A systematic review
Source: BMC Pediatr. 2008 Apr 24;8:14. doi: 10.1186/1471-2431-8-14 (PMC2390545; doi:10.1186/1471-2431-8-14)
Supplement: Additional file 7 — Detailed intervention descriptions. [file 1471-2431-8-14-S7.doc]

**Additional file 7**

**Detailed intervention descriptions.**

| **First author (year)** | **Type of therapy** |
| --- | --- |
| ***Comprehensive physiotherapy programs*** | |
| Bar-Heim (2006) | Adeli suit treatment: The treatment approach included the Adeli suit (from Zvedzda Corporation, Moscow, Russia, and sized in accordance with the anthropometrical measures) and an intensive, well-structured treatment protocol. Treatment was "conducted in accordance with the original Russian protocol that included the following: 1) massage before fitting the suit; 2) passive stretching of all limb muscles; 3) application of the suit by placing the body into proper alignment and restricting limb positions, thereby loading the patient's musculature; and 4) rigorous exercises in the suit, following an individual program based on functional weight-bearing gross motor activities primarily related to locomotion. Each session included walking activities suited to individual abilities, standing up from sitting, playing with a ball while standing, walking on different terrains, jumping on a trampoline, and climbing stairs and ladders."  Neurodevelopmental therapy (NDT): targets "the central nervous and neuromuscular system and 'teaching' the brain to improve motor performance skills and achieve 'as normal function as possible', in view of the specific lesion in the central nervous system." No strict protocol of treatment, rather an orientation to reacting "in real time to the tone and movement patterns of the patient". Individual functional aims and goals were determined and a structured program was set for each child. The program included: passive stretching of lower limb muscles, techniques of reducing spasticity and facilitating more normal patterns of movements while working on motor functions (walking, standing up from sitting and sitting on a bench). |
| Tsorlakis (2004) | NDT: was based on the fundamental and current principles of the approach, as it has evolved more recently. Therapy was individualized for each child's condition and dictated by the child's unique needs. |
| Ketelaar (2001) | Functional physical therapy: "directed at promoting functional skills instead of normalization of movement". Emphasizes the learning of motor abilities that are meaningful to the child's environment and perceived as problematic by the child or the parents. Children practice these motor abilities in functional situations, with the child having an active role in finding solutions for motor problems rather than having the physical therapist's handling result in a solution. Functional goals, in terms of skills, are established with parents and children based on their priorities. Functional activities are assumed to be learned by repetitive practice of goal-related tasks in functional situations. Content of the therapy varies between the children.  Reference group: previous physical therapy regimen, which content varied between the children. 19 of 27 children were treated according a neurophysiological treatment method (NDT or Vojta), with focus on the principle of normalization of motor performance and quality of movement. |
| Bower (2001) | Physiotherapy: was described by each physiotherapist involved and was found to consist of a mixture of muscle stretching, passive corrective manual handling, positioning, including the use of equipment, orthoses and casting as considered necessary, muscle strengthening and active movement in addition to gross motor skill training along developmental and functional lines as considered appropriate by the child's physiotherapist. Treatment was primarily targeted at gross motor abilities and not manual dexterity. There were remarkable similarities in the documented treatment descriptions between the therapists. For half of the children the [general] aims of the therapy were defined, while specific goals were set for the other half. |
| Bower (1996) | Physiotherapy: "eclectic or comprising a mixture of different ingredients considered appropriate by each individual physiotherapist for each individual child and family". In other groups general aims were documented, and in others specific individual and measurable treatment goals were negotiated, assessed and documented. |
| Palmer (1990, 1988) | Physical therapy: focused on motor development, designed to "optimize expression of components of righting and equilibrium believed to be necessary for continued development of gross motor milestones".  The infant stimulation program: learning games, a curriculum designed to "address a broad range of infant developmental domains: 100 explicitly defined and illustrated cognitive, sensory, language, and motor activities of increasing developmental complexity appropriate for children from birth to 3 years. Fine motor activities include puzzles, crayons, form-matching, and block-building tasks". |
| ***Upper extremity treatment*** | |
| Wallen (2007) | Occupational therapy: a broad range of intervention modalities in order to mirror usual clinical practice. The therapy component was not standardized but "determined by treating clinicians to ensure that the intervention appropriate for participants to meet their individual goals. Intervention techniques included those aimed at improving impairment (e.g. stretching, casting, splinting), and enhancing activities (e.g. motor training, environmental modification, practice of specific goal activities)." |
| Law (1997) | Intensive therapy + casting: facilitation and handling by principles of NDT, focus on changing impairments and improving upper-extremity quality of movement. Bivalved fiberglass upper-extremity cast extended from below the elbow to the palm of the hand. The wrist was held in a position of neutral to 10 degrees extension and the thumb and fingers were free so that their movement was not affected.  Regular occupational therapy: focused on task analysis and facilitating changes in functional skills: self-care, feeding and play. |
| Law (1991) | Regular and intensive NDT: All occupational therapists attended a training workshop on the guidelines for therapy. Although principles for intervention were similar for all children, each child's program was dictated by their unique clinical needs. Additional home programs consisted of specific NDT therapy activities.  Casting: Bivalved fiberglass inhibitive upper-extremity cast extended from below the elbow to the palm of the hand, immobilizing the wrist from neutral to 10 degrees extension. The thumb and fingers were not included. Casts were worn for at least 4 hours per day. |
| Hallam (1996) | Prehensile hand treatment: a treatment program intended to advance the children's prehensile and fine motor skills. Prior to the start of therapy parents received a handbook on the importance of hand function in relation to daily life and on ideas and suggestions to help the parents to get involved with the research while helping and playing with their child. The exercises in the handbook were intended as guides to aid purposeful play rather than as definitive routines to be followed rigidly. The therapy program followed the regime described in the handbook given to the parents. At the beginning of each session the child was either placed or requested to sit in a good position, usually in a specially adapted chair or a good small seat with the feet flat on a footrest or the floor. If the child demonstrated marked spasticity that inhibited correct posture or movement, time was spent counteracting it with general physiotherapy before attempting any specific hand therapy. The therapy program included 14 different toys and play equipment: threading cotton reels, shape sorter, balls to roll and catch with bells/chimes inside, stacking humpty dumpty, rocking ring stacker, more difficult shape sorter, cup and spoons for pretend play, cardboard picture books (to practice page turning), stacking beakers, mirror, picture form boards, square blocks for threading and building, balls for color matching and throwing/catching, building blocks.  General physiotherapy: was modeled Bobath NDT, combined with any specific exercises requested by the child's regular therapist to ensure continuity and constancy of treatment. The aims were: "to give the child all possible mobility, to help the child develop without excessive effort which will increase spasticity, to [have] control over their own abnormal sensori-motor patterns with a view to obtaining more normal functional activity." Securing such changes in postural tone and abnormal patterns that lead towards the normal state cannot be achieved unless the degree and distribution of hypertonus is capable of being altered by handling and stimulation. |
| ***Strength training programs*** | |
| Liao (2007) | Loaded sit-to-stand (STS) group: The trainer demonstrated and instructed the loaded STS exercise, provided a body vest and weight to the children and their caregivers, and educated the caregivers to motivate children and to encourage the child to perform as many repetitions as possible. 1 repetition maximum (1-RM) was tested prior to training, and was defined as "the maximal load a child is capable of carrying while standing up one time from a sitting position without falling". Each session consisted of: 1) 5 to 10 minute warm-up activities: active movements of lower extremities, stretching of hip abductors, ankle plantar flexors, hamstring muscles, and lumbar extensors, 2) STS 10 times with body vest at 20% of 1-RM STS load, 3) 1-2 minute rest, 4) STS with the load at 50% 1-RM STS repeatedly without stopping until fatigue, 5) 2-3 minute rest, 6) STS activities again for 10 times with 20% of 1-RM STS weight, and 7) cooling down exercises, similar to warm-up exercises. The height of the chair the child sat in and performed the STS exercises at home was similar for that used for the maximum load of the loaded STS test. The progressive increasing of weight was adjusted to 50% 1-RM STS every 2 weeks according to the latest loaded STS tests. |
| Patikas (2006) | Training: started 3 to 4 weeks after surgery (no longer painful to perform the exercises and no danger of recurring injury). The children were instructed to carry out the training program at least 3 times a week, with an optimal target of 4 times a week. Each session was 30-45 minutes long (depending on the child) and consisted of 7 exercises: 1) pelvis raised lying supine knees flexed at 90º, 2) unilateral knee extension lying supine with the hip flexed at 30º, 3) sit-ups approaching with the hands to the left, center and right, 4) unilateral hip and knee flexion from lying supine, 5) knee flexion from prone position, 6) knee flexion from kneeling position with the trunk in upward position, 7) sitting down and standing up from a chair with the hands projected to the front. Sitting position at 90º of knee and hip flexion. For exercises 1 and 7 the tights fastened together distally with rubber bans to prohibit excessive hip abduction. Two sets of 5 repetitions were performed for each exercise, and for both legs with a 1-minute rest between each set and drill. The movement velocity was 4 to 5 seconds per repletion, including slow return to the initial position in order to evoke eccentric muscle activation. The resistance was progressively increased by gradually eliminating the external support during the exercise. As soon as the children succeed in overcoming the resistance against gravity without assistance, the parents increased further resistance for exercises 2, 4 and 5 using elastic bans. Additional rubber band layers were applied if the child could repeat the whole set without compensatory movements from other muscle groups.  2 physiotherapists taught and supervised the training protocol and gave instructions to the child's parents about executing the exercises following hospital discharge, as well as giving a detailed written description of the exercises. The research team contacted the parents at home by telephone at least twice a month to clarify potential issues related to the training and to learn of possible adverse effects. |
| Unger (2005) | Individually designed circuit training: 1-3 times a week for 8 weeks in school hours. Individually designed in consultation with the children's therapists to ensure correct selection exercises. The training program included a 5-min warm-up on a stationary bicycle and 8-12 exercises (selected for each subject from a 28-station circuit targeting upper and lower limbs and trunk). The circuit was completed at the subject's own pace, with self-selected speed for each exercise. Movements had to be controlled and smooth. Exercises were progressive according to guidelines by McArdle et al. (1996). Initial resistance was set to allow at least one set of 6–10 repetitions. When 3 sets of 12 repetitions were reached resistance was increased and repetitions reduced. This process was repeated as soon as the subject could complete 3 sets of 12 repetitions. Resistance was provided by body weights or free weights (dumbbells, ankle and wrist cuff weights, elastic and rubber bands). |
| Dodd (2003, 2004) | Strength-training: Target muscle groups were ankle plantar flexors, knee extensors and hip extensors. The program included: "1) bilateral heel raises in which the participant stood on the edge of a stable, light-weight portable step (height 20cm) and raised and lowered his or her heels through the full available range, 2) bilateral half squats in which from a standing position, the participant slowly squatted until knees were flexed to between 30 and 60 degrees. A large inflatable ball (55cm diameter) was placed between the lower back of the participant and the wall to help guide and standardize the exercise; 3) step-ups where the participant stepped onto and off portable steps." The training load was adjusted by adding free weights to a backpack worn by the participants to ensure optimal strengthening benefit. Once the initial load was determined, participants were instructed to complete three sets of 8 to 10 repetitions of each exercise, 3 times a week.  Control group: continued normal daily activities, including school and sport. Participants were also able to attend their normal physiotherapy program, provided therapy did not include a progressive resistance exercise program. |
| ***Cardiovascular fitness and aerobic programs*** | |
| Chad (1999) | Physical activity program: Each session: exercise for upper extremities 20min, lower extremities 20 min, truncal region 20 min; exercise focused on the facilitation of normal movement and weight-bearing activities. |
| Van den Berg-Emons (1998) | Physical training: Activities consisted of predominantly aerobic exercises: cycling, wheelchair driving, running, swimming, training on "flying-saucer", and mat exercises. Four times per week above the normal school and therapy program. Therapy program was according to personal needs (varied from 0 to 2.5 hrs/week for all children included in the study). |
| ***Constraint induced therapy*** | |
| Charles (2006) | CI-therapy with a sling: The intervention was provided on 10 out of 12 consecutive days during summer or school vacations (typically 2 weeks of weekdays) with groups of 2–4 children. Children wore a sling on the non-involved upper extremity for the 6 hour-session. After the session the sling was removed. The sling was strapped to the child's trunk and the distal end was sewn shut to prevent use of the non-involved hand. |
| Taub (2004) | CI-therapy with a cast: 2 components: 1) child's less-impaired upper extremity was casted from upper arm to fingertips by using a lightweight fiberglass cast. The cast was bivalved to enable easy weekly removal to check skin integrity and allow range of motion, 2) intensive treatment (shaping) for the involved upper extremity for 6 hours each day for 21 consecutive days. Training procedures: Shaping involved presenting interesting and useful activities to the child that provided immediate rewards. When a new skill emerged the therapist proceeded to shape this by increasing demands in quality. "Tasks such as reaching, grasping, holding, manipulating an object, bearing weight on the arm, and making hand gestures were divided into their small component skills, which were worked on individually and later chained together to comprise a target activity. The CI therapist also incorporated everyday tasks (e.g. dressing/undressing, eating, bathing, and grooming). Parents were encouraged to join in therapy-related activities and encourage their child to use newly acquired skills when the therapist was not present. When a child showed signs of fatigue, frustration, or reduced interest, the therapist adapted the activities but did not cease the therapy. Rest intervals were given as needed." On average, a child participated in at least 2 distinct upper extremity activities each hour. The therapist was responsible for ensuring that the full dose of 6 hours of active treatment per day was provided.  Control group: continued their participation in conventional PT and/or OT, which was established earlier. After 6 weeks the control group crossed over to receive CI-therapy for 21 days. |
| ***Sensorimotor training programs*** | |
| Bumin (2001) | Sensory-perceptual-motor training (SPM) protocol: 1) sensory systems input activities (wheelbarrow hand walk, swimming/drying off); 2) activities for body awareness (window game, body pushing); 3) vestibular system activities (swing, jumping on a trampoline, climbing the wall bar); 4) tactile system activities (stereo gnosis training, textured road); 5) motor planning activities (statue spinning, mystery writing); 6) balance and postural responses activities (balance activities used were: two knees and two hands, two hands and one foot, two elbows and one knee, two knees and kneel hand push); postural responses and ocular control activities (ball catch, two person ball catch, ball foot toss, throwing a ball into a basket and a target); 8) bilateral motor co-ordination and motor planning (Inchworm art, stick ball); 9) visual spatial perception (matching the geometric shapes, puzzle activities); 10) fine motor skills and motor planning (bead stringing, pegboard activities, writing at different positions, tear art on knee position, button up, knotting, design copying); 11) right - left discrimination training; and 12) standing and walking training. Also home program (not specified).  Control group: home training, not specified. |
| ***Balance training*** | |
| Ledebt (2005) | Balance training: static and dynamic tasks on a force plate in 30-minute sessions, 3 sessions per week for 6 weeks (a total of 18 sessions). "The force plate was displayed as a square (40x40cm) on a vertical screen (2.5x2.5m), situated at a distance of 1.3m in front of the child standing at the center of the force plate. The center of pressure was represented by a red dot. The children were required to either keep the dot within a target area located directly in front of them at an eye height that corresponded to the center of the base of support (static task), or to move the dot towards successive positions occupied by the target area (dynamic tasks). Three dynamic tasks were performed: a) a "circle task", in which the target areas appeared at regular distances along a circular path in either a clockwise or counter-clockwise direction, b) a "random task", in which the targets appeared at unpredictable places, and c) a "lateral weight-shifting task", in which the target area moved continuously to and from the center and either to a position to the left or right of the center. During the latter task the distance was gradually increased. The distance was also progressively increased from one trial to the next when the participant was able to reach the most distant located target." The children wore their own shoes including ankle-foot orthoses or insoles that corrected leg-length discrepancies. |
| ***Therapy with animals*** | |
| Benda (2003) | Hippotherapy: 2 trained therapy horses with similar stride lengths, one small and one medium size were selected for the study in order to accommodate both the smaller and larger children. The horse was tacked with a fleece pad and flat surcingle (a belt to secure pad), and the child was mounted on the horse sitting forward astride the fleece. A horse handler led the horse on a designated track at a steady walk for 4 minutes clockwise and 4 minutes counterclockwise. A physical therapist and assistant walked aside the horse but no postural support was provided.  Stationary barrel: made from a 55-gallon drum approximating the girth of horse, was covered with the fleece and mounted on supports at the approximate height of an average horse. A television with VCR was mounted in front of the barrel to encourage the child to maintain forward attention and quiet sitting. The child sat astride the barrel, as he would on a horse with three assistants in identical places to previously. |
| MacKinnon (1995) | Horseback riding: focused on the development of functional riding skills, basic horse and stable knowledge, and skills at games on horseback. Children in the mild group rode using saddles and were encouraged to use reins, holding one in each hand. The children in the moderate group rode on saddle pads with surcingles. |
