# Supplementary material for: Effectiveness of physical therapy interventions for children with cerebral palsy: A systematic review
Source: BMC Pediatr. 2008 Apr 24;8:14. doi: 10.1186/1471-2431-8-14 (PMC2390545; doi:10.1186/1471-2431-8-14)
Supplement: Additional file 8 — Effectiveness of physical therapy interventions by the ICF components. Outcomes with moderate evidence are in bold. [file 1471-2431-8-14-S8.doc]

**Additional file 8**

**Effectiveness of physical therapy interventions by the ICF components.** Outcomes with moderate evidence are in bold. (Table continues)

| *Intervention category* | Part 1: functioning and disability | | Part 2: contextual factors | |
| --- | --- | --- | --- | --- |
| First author (year) | Body functions and structures | Activities and participation | Environmental factors | Personal factors |
| **1) Statistically significant difference in favor of the intervention group** | | | | |
| *Comprehensive physiotherapy programs* | | | | |
| Bar-Haim (2006) | Metabolic cost of stair climbing (10mo) ↑ p=0.0004 |  |  |  |
| Tsorlakis (2004) |  | GMFM-66 ↑ p=0.018, ES=0.8 |  |  |
| Ketelaar (2001) |  | GMFM: standing (6+18mo) ↑ p=0.01, walking, running, jumping (6mo) ↑ p=0.04; PEDI (18mo): functional skills, self-care ↑ p=0.01, mobility↑ p<0.05; caregiver assistance: self-care ↑ p<0.01, mobility ↑ p<0.05 |  |  |
| Palmer (1990, 1988) |  |  | HOME sub item: emotional and verbal responsivity of mother ↑ p=0.04 |  |
| *Upper extremity treatment* | | | | |
| Wallen (2007) | **ROM: active supination ↑ p=0.008** | **GAS ↑ p=0.054** |  |  |
| Law (1991) | ROM: wrist extension ↑ p=0.02* | QUEST (6mo) ↑ p=0.03* |  |  |
| Hallam (1996) |  | **GMDS developmental quotient ↑ p<0.002†** |  |  |
| *Strength training* | | | | |
| Liao (2007) | PCI ↑ p=0.005, ES=1.34 | GMFM-88: standing, walking, running, jumping ↑ p=0.02, ES=1.17; Loaded sit-to-stand test: maximum load ↑ p=0.001, ES=1.78 |  |  |
| Unger (2005) | 3D gait analysis (free speed): sum of ankle, knee and hip angles at midstance ↓ (p value unclear) |  |  | Self-perception: body image ↑ (p value unclear) |
| Dodd (2003, 2004) | Hand-held dynamometer: ankle plantar flexor and knee extensor strength↑ (6wk) p=0.046, (18wk) p=0.041 |  |  |  |
| *Cardiovascular fitness* | | | | |
| Chad (1999) | Femoral neck bone mineral content ↑ p=0.03, density ↑ p=0.02 |  |  |  |
| Van Den Berg-Emons (1998) | Peak aerobic power ↑ (9mo) p=0.05;  Fat mass↓ p<0.05 |  |  |  |
| *Constraint-induced therapy* | | | | |
| Charles (2006) |  | **Caregiver Functional Use Survey: hand use:**  **frequency ↑ (1wk) p<0.01, ES=0.3**  **quality ↑ (1+6mo) p<0.01, ES=0.2;** Jebsen-Taylor Test of Hand Function: time to complete tasks ↓ (1wk) p=0.01, ES=0.3; BOTMP: speed, dexterity ↑ (1wk) p=0.005, ES=0.4 |  |  |
| Taub (2004) |  | Emerging Behaviours Scale↑ p<0.0001; Pediatric Motor Activity Log: **frequency and quality of hand use ↑ (3wk) p<0.0001** |  |  |
| *Balance training* | | | | |
| Ledebt (2005) |  | Force Plate: displacement ↓ forward p=0.01, backward p=0.006 Leaning ↓ forward p=0.003, backward p=0.001, paretic side p=0.022 and non-paretic side p=0.001; step length of non-paretic leg ↑ p=0.017 |  |  |
| *Therapy with animals* | | | | |
| MacKinnon (1995) |  | PDMS-FM sub item: grasping ↑ p=0.045 |  |  |
| **2) Statistically significant difference in favor of the control group** | | | | |
| Palmer (1990, 1988) |  | Attained motor skills: independent walking p=0.01; BSID: motor ↑ (6mo)p=0.02, (12mo) p<0.01; mental ↑ (6mo) p=0.05 |  |  |
| Dodd (2003, 2004) |  |  |  | **Self-perception profile for children: scholastic competence ↑ (6wk) p=0.04, (18wk) p=0.016, social acceptance ↑ (18wk) p=0.03** |
| **3) No statistically significant differences between the groups** | | | | |
| *Comprehensive PT* | | | | |
| Bar-Haim (2006) |  | GMFM-66 |  |  |
| Tsorlakis (2004) |  | GMFM-88 (16wk) |  |  |
| Ketelaar (2001) |  | GMFM: walking, running, jumping (18mo); PEDI (6mo) |  |  |
| Bower (2001) |  | GMFM, GMPM |  |  |
| Bower (1996) |  | GMFM |  |  |
| Palmer (1990, 1988) |  | Attained motor skills; BSID (12mo): mental ; VABS social quotient | HOME total, mother–child relationship | Infant temperament |
| *Upper extremity treatment* | | | | |
| Wallen (2007) | Tardieu scale: spasticity;  ROM: passive elbow | PEDI; QUEST; COPM; MA; CHQ |  |  |
| Law (1997) |  | PDMS-FM; QUEST; COPM |  |  |
| Law (1991) |  | PDMS-FM; QUEST (9mo) |  |  |
| Hallam (1996) |  | GMDS: chronological and mental age |  |  |
| *Strength training* | | | | |
| Liao (2007) | Knee extensor strength | **Gait speed** |  |  |
| Patikas (2006) | MAS; Muscle tone; knee extension, flexion and ROM; oxygen consumption; energy expenditure | GMFM: standing, walking, running, jumping Gait analysis: e.g. **stride length, gait speed** |  |  |
| Unger (2005) | 3D gait analysis: ankle, knee and hip angles separately at midstance, knee angle at heel strike | **3D gait analysis: velocity, stride length,** cadence |  | Self-perception: functional competence |
| Dodd (2003, 2004) | Hand-held dynamometer: combined ankle plantar flexor, knee and hip extensor strength | GMFM, **gait speed**, timed stair test |  | Self-perception: athletic competence, physical appearance, behavioral conduct and global self-worth |
| *Cardiovascular fitness* | | | | |
| Chad (1999) | Proximal femur bone mineral content |  |  |  |
| Van Den Berg-Emons (1998) | Peak anaerobic and mean aerobic power | Physical activity |  |  |
| *Constraint-induced therapy* | | | | |
| Charles (2006) | Sensibility; hand-grip force; shoulder, elbow and wrist muscle tone | Hand function (1+6mo); BOTMP: speed and dexterity (1+6mo) |  |  |
| Taub (2004) |  | QUEST |  |  |
| *Balance training* | | | | |
| Ledebt (2005) |  | Force plate (quiet stance): time on target, displacement toward paretic and non paretic sides; step length of paretic leg |  |  |
| *Therapy with animals* | | | | |
| Benda (2003) | Muscle asymmetry |  |  |  |
| MacKinnon (1995) | Sitting posture‡ | GMFM; PDMS-FM total; VABS-ADL: socialization; CBC; BOTMP |  | HSPC |

*For the two casted arm groups compared to two uncasted arm groups.

†For the prehensile hand treatment+NDT and extra NDT groups compared to NDT group.

‡ by a scale developed by Bertoti DB: Therapeutic riding conferences-Positive progress. In Proceedings of the 6th International Therapeutic Riding Congress; August 23-27; Toronto, Ontario. 1988: 400-405.

↑=improvement or increase of the outcome, ↓=deterioration or decrease of the outcome, mo=months, wk=weeks, ADL=activities of daily living, BSID=Bayley Scales of Infant Development, BOTMP=Bruininks-Oseretsky Test of Motor Proficiency, CBC=Child Behaviour Checklist, ES=effect size, GMDF=Griffith's Mental Developmental Scales, GMFM = Gross Motor Function Measure, HOME=Home Observation for Measurement of the Environment, HSPC=Harter Self-perception Profile for Children, MA=Melbourne assessment of unilateral upper limb function, MAS=Modified Asworth Scale, MPOC=Measure of Processes of Care, PCI=Physiological Cost Index, PEDI=Pediatric Evaluation of Disability Inventory, PDMS-FM=Peabody Developmental Motor Scales Fine Motor , QUEST=Quality of Upper Extremity Skills Test, ROM=range of motion, mo=months, VABS=Vineland Adaptive Behavior Scale.
