# Supplementary material for: Effectiveness of physical therapy interventions for children with cerebral palsy: A systematic review
Source: BMC Pediatr. 2008 Apr 24;8:14. doi: 10.1186/1471-2431-8-14 (PMC2390545; doi:10.1186/1471-2431-8-14)
Supplement: Additional file 9 — Full details of the baseline values and changes on all measured outcomes of each trial. [file 1471-2431-8-14-S9.doc]

**Additional file 9**

Full details of the baseline values and changes on all measured outcomes of each trial. (Table continues)

| **First author, year;**  **length of intervention (follow-up)** | **Outcome measure** | **Intervention group** | | **Control group** | | **Statistical difference between the groups** |
| --- | --- | --- | --- | --- | --- | --- |
|  |  | **Mean baseline value (SD)** | **Mean change from baseline (SD)** | **Mean baseline value (SD)** | **Mean change from baseline (SD)** |  |
| ***Comprehensive physiotherapy programs*** | | | | | | |
| **Bar-Haim 2006** |  | **I: NDT with Adeli suit** | | **C: NDT** | |  |
| 4 wk (1mo, 10mo) | **1) GMFM-66** | 54.0 (SEM 4.0) | 1mo: 55.0 (SEM 4.1) 10mo: 54.7 (SEM 4.0) | 52.2 (SEM 3.0) | 1mo: 52.9 (SEM 3.0) 10mo: 54.1 (SEM 3.1) | NS |
|  | **2) Metabolic cost of stair climbing** (mechanical efficiency index, units are 100xkg.m per beat) | 12.7 (SEM 3.5) | 1mo: 15.1 (SEM 4.1) 10mo: 19.6 (SEM 5.3) | 11.1 (SEM 5.0) | 1mo: 12.5 (SEM 5.3) 10mo: 13.1 (SEM 5.4) | 10 mo: I > C (p=.0004) |
| **Tsorlakis 2004** |  | **I: Intensive NDT (5xwk)** | | **C: NDT (2xwk)** | |  |
| 16 wk | **1) GMFM -88** (% points, scale 0-100), Total score | 77.36 (15.89) [range 44.43-97.07] | 16wk: 2.63 | 80.31 (15.15) [range 52.46-98.65] | 16wk: 1.69 | NA |
|  | **2) GMFM -66** (% points, scale 0-100), Total score | 62.17 (12.24) [range 44.03-84.05] | 16wk: 2.36*, Paired t-test, t=5.433 (P<0.001) | 65.85 (14.47) [range 45.91-87.99] | 16wk: 1.18*; Paired t-test, t = 4.449 (p<0.001) | I > C (Cohen's d effect size 0.8, p=.018) |
|  | **3) Improvement in GMFM -88** (score over 1.825 clinically significant) |  | 10 out of 17 |  | 7 out of 17 | NA |
| **Ketelaar 2001** |  | **I: Functional PT (n=28)** | | **C: Previous PT continued (n=27)** | |  |
| 6 mo (12mo, 18mo) | **1) GMFM** (% points, scale 0-100) | | | | | |
|  | - Standing | 82.8 (15.7) | 6mo: 3.1 12mo: 5.7 18mo: 7.8 | 81.2 (20.3) | 6mo: 5.9 12mo: 6.4 18mo: 9.6 | 6 mo: I > C (p=.01) 18mo: I > C (p=.01) |
|  | - Walking, running, jumping | 70.2 (18.2) | 6mo: 6.5 12mo: 13.9 18mo: 16.3 | 70.8 (24.4) | 6mo: 5.5 12mo: 11.3 18mo: 14.0 | 6mo: I > C (p=.04) 18mo: NS |
|  | **2) PEDI functional skills** (scale 0-100) | | | | | |
|  | - Self-care | 68.3 (14.9) | 6mo: 3.6  12mo: 8.4 18mo: 11.4 | 67.3 (10.1) | 6mo: 3.0 12mo: 4.4 18mo: 9.2 | 6mo: NS 18mo: I > C (p=.01) |
|  | - Mobility | 78.2 (11.3) | 6mo: 2.2 12mo: 7.9 18mo: 9.9 | 75.8 (11.6) | 6mo: 0.9 12mo: 4.1 18mo: 5.4 | 18mo: I > C (p<.05) |
|  | **3) PEDI caregiver assistance** | | | | | |
|  | - Self-care | 58.7 (13.7) | 6mo: 4.3 12mo: 12.7 18mo: 15.2 | 59.2 (11.6) | 6mo: 1.4 12mo: 7.3 18mo: 9.1 | 6mo: NS 18mo: I > C (p<.01) |
|  | - Mobility | 72.7 (13.7) | 6mo: 6.1 12mo: 13.7 18mo: 16.0 | 74.0 (15.7) | 6mo: 3.7 12mo: 7.9 18mo: 10.4 | 6mo: NS 18mo: I > C (p<.05) |
| **Bower 2001** |  | **I: Aim-directed therapy (n=28)** | | **C: Goal-directed therapy (n=27)** | |  |
| 6 mo (9mo, 12 mo, 18mo) | **1) GMFM total score** (% points, scale 0-100) | No data | 12 mo: 4.4 | No data | 12 mo: 4.6 | NS |
|  | **2) GMPM** total score (% points, scale 0-100) | No data | 6 mo: 2.9 | No data | 6 mo: 1.8 | NS |
|  | **3) MPOC** (scale 0-7) | | | | | |
|  | - Enabling and partnership | 5.4 (1.5) | 6 mo: 0.1 12mo: -0.3 | 5.7 (1.4) | 6mo: 0 12mo: -0.2 | NA |
|  | - Providing general information | 3.0 (1.6) | 6 mo: 0,5 12mo: 0.6 | 3.4 (2.0) | 6mo: 0,5 12mo: 0.4 | NA |
|  | - Providing specific information | 5.3 (1.6) | 6 mo: 0,1 12mo: -0.3 | 5.4 (1.6) | 6mo: 0,2 12mo: -0.3 | NA |
|  | - Coordinated comprehensive care | 6.0 (1.2) | 6 mo: -0,1 12mo: -0.2 | 5.5 (1.5) | 6mo: 0,3 12mo: 0 | NA |
|  | - Respectful and supportive care | 5.7 (1.4) | 6 mo: 0,1 12mo: -0.1 | 6.0 (1.3) | 6mo: 0,1 12mo: -0.2 | NA |
| **Bower 1996** |  | **I: Conventional PT+aims (n=11)** | | **C1: Intensive PT+aims (n=11)** | |  |
| 2 wk | **1) GMFM** (Number of aims set and improved /deteriorated >1.825% points) | No of aims set | No of aims improved/deteriorated | No of aims set | No of aims improved/deteriorated | NS |
|  | - Lying & rolling | 2 | 0/1 | 3 | 2/0 |  |
|  | - Sitting | 4 | 2/2 | 7 | 4/1 |  |
|  | - Crawling & kneeling | 1 | 0/1 | 0 | 0/0 |  |
|  | - Standing | 4 | 1/3 | 5 | 3/0 |  |
|  | - Walking, running, jumping | 2 | 1/0 | 4 | 2/0 |  |
|  | - Total score (% points, SD) | 36.3 (17.9) | 2 | 31.9 (21.5) | 2 |  |
|  |  | **C2: Conventional PT+goals (n=11)** | | **C3: Intensive PT+goals (n=11)** | |  |
|  | **1) GMFM** (Number of aims set and improved /deteriorated >1.825 % points) | No of goals set | No of goals improved/deteriorated | No of goals set | No of goals improved/deteriorated | NS |
|  | - Lying & rolling | 1 | 1/0 | 4 | 3/1 |  |
|  | - Sitting | 9 | 8/1 | 7 | 6/1 |  |
|  | - Crawling & kneeling | 0 | 0/0 | 2 | 1/0 |  |
|  | - Standing | 3 | 2/1 | 6 | 5/0 |  |
|  | - Walking, running, jumping | 2 | 1/0 | 2 | 1/0 |  |
|  | - Total score (% points, SD) | 32.4 (16.2) | 2 | 39.8 (21.2) | 4 |  |
| **Palmer 1990, 1988** |  | **I: NDT** | | **C: Infant stimulation + NDT** | |  |
| 12 mo | **1) HOME** (45 items) | | | | | |
|  | - Emotional and verbal responsivity of mother | 8.16 | 12mo: 1.20 | 8.87 | 12mo: 0.32 | I > C p=0.04 (95% CI 0.13 to 1.63) |
|  | - Avoidance of restriction and punishment | 5.88 | 12mo: 0.0 | 6.00 | 12mo: -0.18 | NS |
|  | - Organization of the physical and temporal environment | 5.08 | 12mo: -0.12 | 5.13 | 12mo: -0.18 | NS |
|  | - Provision of appropriate play materials | 5.88 | 12mo: 1.88 | 5.78 | 12mo: 1.86 | NS |
|  | - Maternal involvement with child | 4.2 | 12mo: 0.6 | 4.65 | 12mo: 0.14 | NS |
|  | - Opportunities for variety in daily stimulation | 2.64 | 12mo: 1.0 | 2.96 | 12mo: 0.64 | NS |
|  | - Total score | 31.8 | 12 mo:3.32 | 33.39 | 12mo: 2.59 | NS |
|  | **2) The Mother-Child Relationship Evaluation** (48 items) | | | | | |
|  | - Acceptance | 40.12 | 12mo: 1.0 | 41.83 | 12mo: 2.14 | NS |
|  | - Overprotection | 36.76 | 12mo: -1.52 | 35.65 | 12mo: -2.00 | NS |
|  | - Overindulgence | 33.52 | 12mo: -1.20 | 33.09 | 12mo: -1.32 | NS |
|  | -Rejection | 35.68 | 12mo: 0.12 | 33.35 | 12mo: 0.77 | NS |
|  | **3) Carey Infant Temperament questionnaire** | | | | | |
|  | - Activity | 3.04 | 12mo: 0.08 | 3.70 | 12mo: 0.64 | NS |
|  | - Rhythmicity | 3.72 | 12mo: 0.24 | 3.43 | 12mo: -0.36 | NS |
|  | - Adaptability | 6.84 | 12mo: -1.52 | 6.57 | 12mo: -1.36 | NS |
|  | - Approach | 4,92 | 12mo: -0.36 | 4.39 | 12mo: -0.59 | NS |
|  | - Threshold | 8.20 | 12mo: -0.04 | 7.35 | 12mo: 0.09 | NS |
|  | - Intensity | 11.12 | 12mo: 2.00 | 12.04 | 12mo: 0.14 | NS |
|  | - Mood | 8.00 | 12mo: -0.64 | 7.22 | 12mo: -0.64 | NS |
|  | - Distractibility | 4.00 | 12mo: 0.44 | 3.17 | 12mo: 0.27 | NS |
|  | - Persistence | 3.72 | 12mo: 0.28 | 3.91 | 12mo: -0.86 | NS |
|  | **4) Bayley Scales of Infant Development** | | | | | |
|  | - Motor quotient | 53.0 (8.5) | 6mo: -3.8 12mo: -5.0 | 53.0 (9.4) | 6mo: +5.1 12mo: +9.6 | 6mo: C > I (95% CI -16.2 to -1.7, p=0.02) 12mo: C > I (95% CI -24.2 to -5.1, p<0.01) |
|  | - Mental quotient | 62.0 (15.6) | 6mo: +3.6 12mo: +5.0 | 66.1 (18.3) | 6mo: +9.4 12mo: +9.0 | 6mo: C > I (95% CI -11.5 to -0.1, p=0.05) 12mo: NS |
|  | **5) VABS** | | | | | |
|  | - Social quotient | 60.9 (15.4) | 6mo: 67.8 (19.0) 12mo: 67.5 | 65.2 (17.1) | 6mo:  12mo: | 6mo: NS 12mo: NS |
|  | **6) Attained motor skills** (% of children attaining skill) | | | | | |
|  | - Roll from supine to prone position | 83 | 6mo: 100 12mo: 100 | 87 | 6mo: 95 12mo: 100 | NS |
|  | - Sit tripod | 70 | 6mo: 92 12mo: 100 | 78 | 6mo: 96 12mo: 100 | NS |
|  | - Sit alone | 61 | 6mo: 83 12mo: 92 | 65 | 6mo: 91 12mo: 91 | NS |
|  | - Creep in prone position | 56 | 6mo: 96 12mo: 100 | 56 | 6mo: 96 12mo: 95 | NS |
|  | - Crawl on hands and knees | 9 | 6mo: 56 12mo: 88 | 9 | 6mo: 65 12mo: 77 | NS |
|  | - Come to sitting position | 0 | 6mo: 54 12mo: 80 | 0 | 6mo: 61 12mo: 86 | NS |
|  | - Pull to standing position | 17 | 6mo: 62 12mo: 92 | 4 | 6mo: 78 12mo: 86 | NS |
|  | - Cruise | 9 | 6mo: 58 12mo: 84 | 9 | 6mo: 74 12mo: 86 | NS |
|  | - Walk with one hand held | 0 | 6mo: 33 12mo: 52 | 0 | 6mo: 48 12mo: 77 | NS |
|  | - Walk independently | 0 | 6mo: 12 12mo: 36 | 0 | 6mo: 35 12mo: 73 | 6mo: NS 12mo: C > I (p=0.01) |
|  | - Walk backward | 0 | 6mo: 4 12mo: 20 | 0 | 6mo: 4 12mo: 45 | NS |
| ***Upper extremity treatment*** | | | | | | |
| **Wallen 2007** |  | **I: OT** | | **C: No extra OT** | |  |
| 12 wk (2 wk, 3mo, 6mo) | **COPM** (scale 0-10) **-**Performance scores | 3.5±1.3 | 3mo: 2.1±1.7 (95% CI 1.2-3.0) 6mo: 2.7±1.8 (95% CI 1.8-3.6) | 3.2±0.7 | 3mo: 1.2±1.2 (95% CI 0.6-1.8 6mo: 1.7±1.5 (95% CI 0.8-2.6) | NS |
|  | -Satisfaction scores | 3.6±1.5 | 3mo: 2.5±1.9 (95% CI 1.6-3.5) 6mo: 3.3±2.2 (95% CI 2.2-4.5) | 4.0±2.1 | 3mo: 1.4±1.4 (95% CI 0.6-2.1 6mo: 2.1±1.7 (95% CI 1.1-3.2) | NS |
|  | **Goal Attainment Scale** (normalized T scores, whereby a score of 50 means that goals, on average, are achieved) | Goals identified | 3mo: 42.2±10.6 (95% CI 26.8-47.7) 6mo: 51.4±11.1 (95% CI 45.7-57.1) | Goals identified | 3mo: 32.9±10.3 (95% CI 27.2-38.7) 6mo: 51.6±12.0 (95% CI 33.4-47.9) | 3mo: NA 6mo: I>C (p=.054) |
|  | **Melbourne Assessment of unilateral upper limb function** | No data | 3mo: No data 6 mo: +3.9* | No data | 3mo: No data 6 mo: No data | NS |
|  | **QUEST** (dissociated movement, grasp, protective extension, weight bearing, scale -12 to 106) | No data | No data | No data | No data | NS |
|  | **PEDI** (scale 0-100) -Functional skills -Caregiver assistance | No data | No data | No data | No data | NS |
|  | **CHQ** | No data | No data | No data | No data | NS |
|  | **Tardieu scale** (Proportion of potentially available change of spasticity at each muscle group that was actually achieved) |  |  |  |  |  |
|  | - Elbow flexors | No data | 2we: -4.8±42.6 (95% CI -29.5-19.8) 3mo: -3.0±26.2 (95% CI -17.5-11.5) 6mo: -12.7±86.9 (95% CI 59.0-33.6) | No data | 2we: -15.0±89.6 (95% CI -79.1-49.1) 3mo: 28.2±29.9 (95% CI 10.9-45.4) 6mo: 28.1±36.6 (95% CI 4.8-51.4) | NS |
|  | - Pronators | No data | 2we: -12.3±37.5 (95% CI-33.1-8.4) | No data | No data | NS |
|  | **ROM** passive elbow flexors and pronators | No data | No data | No data | No data | NS |
|  | **ROM** active supination | No data | 6mo: +1.5 | No data | 6mo:-19.5 | I > C (p=.008) |
|  | **Parent questionnaire** (rating of the child's arm compared with baseline: much worse, a bit worse, much the same, a bit better, much better) | - | 2we:  3mo:  6mo: | - | 2we:  3mo:  6mo: | NA |
| **Law 1997** |  | **I: Intensive NDT+casting** | | **C: Regular OT** | |  |
| 4 mo | **1) PDMS-FM** | 20.4 (9.0) | 4mo: 1.4 | 19.2 (8.6) | 4mo: 1.7 | NS |
|  | **2) QUEST** | 51.3 (22.3) | 4mo: 2.0 | 41.5 (25.3) | 4mo: 5.8 | NS |
|  | **3) COPM performance scores** | 3.2 (1.5) | 4mo: 3.3 | 3.4 (1.0) | 4mo: 2.3 | NS |
| **Law 1991** |  | **I: Intensive NDT+ cast** | | **C1: Intensive NDT** | |  |
| 6 mo (9 mo) | **1) PDMS-FM** (age equivalent in mo) | 30.3 (13.2) | 6mo: 5.26 | 25.0 (17.5) | 6mo: 3.11 | NS |
|  |  |  | 9mo: 6.33 |  | 9mo: 3.24 | NS |
|  | **2) QUEST** (percentage score) | 61.9 (21.9) | 6mo: 4.89 | 47.1 (26.4) | 6mo: 0.8 | I, C2 > C1, C3 (p=0.03) |
|  |  |  | 9mo: 4.1 |  | 9mo: 1.55 | NS |
|  | **3) ROM** (wrist extension) | No data | No data | No data | No data | I, C2 > C1, C3 (p=0.02) |
|  |  | **C2: Regular NDT + cast** | | **C3: Regular NDT** | |  |
|  | **1) PDMS-FM** (age equivalent in mo) | 30.6 (18.4) | 6mo: 3.05 | 27.3 (20.3) | 6mo: 3.44 |  |
|  |  |  | 9mo: 2.44 |  | 9mo: 4.94 |  |
|  | **2) QUEST** (percentage score) | 43.9 (25.7) | 6mo: 7.02 | 45.8 (29.6) | 6mo: 1.34 |  |
|  |  |  | 9mo: 6.11 |  | 9mo: 1.47 |  |
|  | **3) ROM** (wrist extension) | No data | No data | No data | No data |  |
| **Hallam 1996** |  | **I: prehensile hand treatment + NDT** | | **C1: NDT 2xweek** | |  |
| 6mo | **1) GMDS** |  |  |  |  |  |
|  | - Chronological age in mo | 18.3 (.352) | 6mo: +6 | 18.3 (.387) | 6mo: -6.1 | NS |
|  | - Mental age in mo | 12.1 (4.58) | 6mo: +5.1 | 12.5 (4.29) | 6mo: +5.6 | NS |
|  | - Locomotor score | 60 (23.2) | 6mo: +3 | 59 (24.3) | 6mo: +9 | I, C1 > C2 (p<0.000) |
|  | - Personal-social score | 73 (25.6) | 6mo: 0 | 78 (22.7) | 6mo: +4 | C1 > C2 (p<0.0010) |
|  | - Hearing-speech score | 73 (21.8) | 6mo: +5 | 76 (21.3) | 6mo: +6 | I, C1 > C2 (p<0.005) |
|  | - Eye-hand co-ordination score | 67 (25.6) | 6mo: +1 | 64 (28.4) | 6mo: +7 | I, C1 > C2 (p<0.001) |
|  | - Performance | 63 (25.7) | 6mo: +8 | 67 (24.6) | 6mo: +9 | I, C1 > C2 (p<0.003) |
|  | - Total: developmental quotient (average of all sub-quotients) | 66 (23.4) | 6mo: +5 | 68 (21.1) | 6mo: +7 | I, C1 > C2 (p<0.002) |
|  | **2) Hand-grip force** (Dynamometer) | **(n=21)** |  | **(n=17)** |  |  |
|  | - Median peak power | No data | No data | No data | No data | NA |
|  |  | **C2: NDT 1xweek** | |  | | |
|  | **1) GMDS** |  |  |  |  |  |
|  | - Chronological age in mo | 18.4 (.392) | 6mo: +5.9 |  |  |  |
|  | - Mental age in mo | 11.3 (4.56) | 6mo: +1.6 |  |  |  |
|  | - Locomotor score | 50 (28.7) | 6mo: -9 |  |  |  |
|  | - Personal-social score | 65 (25.1) | 6mo: -4 |  |  |  |
|  | - Hearing-speech score | 68 (27.4) | 6mo: -8 |  |  |  |
|  | - Eye-hand co-ordination score | 51 (28.1) | 6mo: -3 |  |  |  |
|  | - Performance | 57 ( 24.8) | 6mo: -5 |  |  |  |
|  | - Developmental quotient (average of all sub-quotients) | 58 (24.1) | 6mo: -6 |  |  |  |
|  | **2) Hand-grip force** (Dynamometer) | **(n=13)** |  |  |  |  |
|  | - Median peak power | No data | No data |  |  |  |
| ***Strength training programs*** | | | | | | |
| **Liao 2007** |  | **I: Home-based Loaded sit-to stand exercise** | | **C: No extra exercise** | |  |
| 6 wk | **1) GMFM-88, dimensions D, E** | 76.6±4.4 | +3.2 | 83.1±3.2 | +0.4 | I > C (Effect size 1.17, p=0.02) |
|  | **2) Gait speed** (m/min, self selected speed, 10m distance) | 56.9±5.1 | +1.5 | 63.8±3.0 | -1.8 | NS |
|  | **3) Maximum load of the loaded sit-to-stand test** (kg, 1-RM of the max load the child is capable of carrying while standing up 1 time from a sitting position without falling) | 9.6±1.6 | +3.9 | 11.3±1.8 | +0.9 | I > C (Effect size 1.78. p=0.001) |
|  | **4) Maximum knee extensor strength** (average torque of 3 separate trials of both legs) | 5.3±0.8 | +0.7 | 5.7±1.1 | +0.7 | NS |
|  | **5) PCI** (difference between the resting and walking heart rates divided by the walking speed) | 1.14±0.14 | -0.13 | 1.02±0.09 | +0.05 | I > C (Effect size 1.34, p=0.005) |
| **Patikas 2006 a,b** |  | **I: Strength training + PT** | | **C: No training + PT** | |  |
| 9 mo | **1) MAS** (0-4 nominal scale) | No data | 6mo: 95% CI -1.1 to -0.3* 12mo: 95% CI -1.1 to -0.3* | No data | 6mo: 95% CI -1.0 to -0.2* 12mo: 95% CI -1.0 to -0.3* | NS |
|  | **2) Knee extension deficit** (º) | -5.0±9.8 | 6mo: -1.1±4.4 12mo: -1.1±4.7 | -5.0±8.3 | 6mo: -0.3±6.6* 12mo:-0.5±8.6 | NS |
|  | **3) Knee flexion** (º) | No data | 6mo: 95%CI -14.2 to 2.1 12mo: 95%CI -12.3 to -0.0* | No data | 6mo: 95% CI -19.4 to -3.6* 12mo: 95% CI -12.4 to -0.4* | NS |
|  | **4) Knee ROM** (º) | No data | 6mo: 95% CI -10.9 to 6.7 12mo: 95% CI -9.7 to 5.2 | No data | 6mo: 95% CI -15.4 to 1.9 12mo: 95% CI -9.1 to 5.4 | NS |
|  | **5) GMFM** (% points) | No data |  | No data |  | NS |
|  | - Standing (% of maximum) | No data | 6mo: no data 12mo: 95%CI -8.5 to 5.0 | No data | 6mo: no data 12mo: 95%CI 6.1 to 19.3* | NS |
|  | - Walking, running, jumping (% of maximum) | No data | 6mo: no data 12mo: 95%CI -13.0 to -0.4* | No data | 6mo: no data 12mo: 95%CI -5.5 to 6.9 | NS |
|  | - Total score | No data | 6mo: no data 12mo: 95%CI -4.8 to 1.1 | No data | 6mo: no data 12mo: 95%CI 0.7 to 6.5* | NS |
|  | **6) Oxygen consumption** | No data | No data | No data | No data | NS |
|  | **7) Energy expenditure index (heart beats/min)** | No data | No data | No data | No data | NS |
|  | **8) Gait analysis**  - Walking speed (cm/s), stance phase duration (% of gait cycle), stride duration (s), stride length (cm), normalcy index, max. hip extension, min. knee flexion during terminal swing, max. plantarflexion during stance-swing transition, max. hip power absorption (W/kg), max. knee power absorption during loading response (W/kg), max. plantarflexion moment (Nm/kg), max. plantarflexion power generation (W/kg) | No data | No data | No data | No data | NS |
| **Unger, 2005** |  | **I: Circuit training** | | **C: No training** | |  |
| 9 wk | **1) 3D gait analysis (free speed)** | | | | | |
|  | - Knee angle at mid-stance phase (º) | 19.3 (10.1) | -1.5 | 19.1 (5.5) | +0.1 | NS |
|  | - Ankle angle at mid-stance phase (º) | -8.6 (6.1) | +0.9 | -9.6 (2.2) | -1.4 | NS |
|  | - Hip angle at mid-stance phase (º) | 20.1 (8.9) | -1.7 | 14.6 (7.7) | +1.2 | NS |
|  | - Sum of ankle, knee and hip angles at mid-stance (º) | 49.7 (16.9) | -4.9 | 43.4 (14.3) | +2.6 | I > C (p value unclear) |
|  | - Knee angle at heel strike (º) | 26.7 (6.6) | -1.3 | 26.6 (6.7) | -1.4 | NS |
|  | - Velocity (mm/s) | 1075.6 (235.4) | +43.7 | 1128 (132.0) | +43.4 | NS |
|  | - Stride length (mm/s) | 1111.9 (207.3) | +17.5 | 1112.8 (149.2) | +31.1 | NS |
|  | - Cadence (steps/min) | 114.6 (15.1) | +2.3 | 119.2 (11.6) | +3.9 | NS |
|  | **2) Self-perception** (questionnaire, 11 items) | | | | | |
|  | - Body image (composite score/30) | 23.9 (4.1) | +2 | 23.2 (4.6) | -0.9 | I > C (p value unclear) |
|  | - Functional competence (composite score/25) | 19.9 (3.4) | +1.4 | 19.3 (3.2) | +1.5 | NS |
| **Dodd 2003, 2004** |  | **I: Home-based strength-training** | | **C: Normal daily activity** | |  |
| 6 wk (18w) | **1) Hand-held dynamometer** (Nicholas Manual Muscle Test), kg | | | | | |
|  | - Ankle plantar flexors | 11 (15.8) | 6wk: 0.1 18wk: 5.6 | 17.5 (13.1) | 6wk: -2.1 18wk: -3.7 | NA |
|  | - Knee extensors | 27.5 (10.9) | 6 wk: 5.6 18wk: 5.0 | 23.7 (11.5) | 6wk: 1.8 18wk: 1.5 | NA |
|  | - Hip extensors | 7.9 (7.6) | 6wk: 2.7  18wk: 2.9 | 8.5 (8.4) | 6wk: 3  18wk: 2.1 | NA |
|  | - Ankle plantar flexion+knee extensors | 38.5 (23.2) | 6wk: 5.7*  18wk: 10.7* | 41.1 (20.0) | 6wk: -0.2  18wk: -2.2 | 6wk: I > C (p=.046)  18wk: I > C (p=.041) |
|  | - Total extensors (combined ankle plantar flexor knee extensor, and hip extensor strength) | 46.5 (29.6) | 6wk: 8.3*  18wk: 13.5* | 49.6 (25.9) | 6wk: 2.8  18wk: -0.1 | NS |
|  | **2) GMFM** (results are presented in % of 13 items, scale 0-100) | | | | | |
|  | - Standing | 75.2 (14.4) | 6wk: 4.9  18wk: 5.2 | 74.6 (20.9) | 6wk: 5.9  18wk: 6.1 | NS |
|  | - Running, walking, jumping | 52.8 (31.3) | 6wk: 4.4  18wk: 5.4 | 68.3 (30.1) | 6wk: 1.2  18wk: -0.5 | NS |
|  | - Sum score of standing and running, walking, jumping | 64.2 (27.8) | 6wk: 4.8  18wk: 5.4 | 71.7 (24.9) | 6wk: 3.6  18wk:2.6 |  |
|  | **3) Self-selected walking speed** (standardized instructions, m/min) | 47.4 (23.3) | 6wk: 0.6  18wk: 1.2 | 49.5 (24.5) | 6wk: 1.0  18wk: 1.9 | NS |
|  | **4) Timed stair test** (s) | 27.4 (34.7) | 6wk: -6.3*  18wk: -2.3 | 22.4 (20.5) | 6wk: -0.7  18wk: -2.7 | NS |
|  | **5) Self-Perception Profile for Children (**scale 0-4) | | | | | |
|  | - Scholastic competence | 3.33 (0.32) | 6wk: -0.23  18wk: -0.16 | 2.57 (0.7) | 6wk: 0.29*  18wk: 0.32* | 6wk: C > I (p=.04)  18wk: C > I (p=.016) |
|  | - Social acceptance | 3.22 (0.79) | 6wk: -0.09  18wk: -0.01 | 2.72 (0.62) | 6wk: 0.32*  18wk: 0.64* | NS  18wk: C > I (p=.03) |
|  | - Athletic competence | 2.46 (0.8) | 6wk: 0.07  18wk: -0.05 | 2.38 (0.81) | 6wk: 0.33*  18wk: 0.45* | NS |
|  | - Physical appearance | 3.25 (0.63) | 6wk: 0.17  18wk: 0.02 | 3.26 (0.6) | 6wk: -0.17  18wk: 0.04 | NS |
|  | - Behavioral conduct | 3.42 (0.45) | 6wk: 0.21  18wk: 0.33 | 2.97 (0.59) | 6wk: 0.04  18wk: 0.11 | NS |
|  | - Global self-worth | 3.41 (0.38) | 6wk: 0.14  18wk: 0.16 | 3.27 (0.52) | 6wk: 0.06  18wk: 0.14 | NS |
| ***Cardiovascular fitness and aerobic programs*** | | | | | | |
| **Chad 1999** |  | **I: Physical activity program** | | **C: No program** | |  |
| 8 mo | **1) Proximal femur BMC** (g) | 8.55 (1.32) | 8mo: 0.98*, 11.5 % | 6.79 (0.59) | 8mo: 0.24, 3.5 % | NS |
|  | **2) Femoral neck BMC** (g) | 1.57 (0.18) | 8mo: 0.15*, 9.6 % | 1.37 (0.10) | 8mo: -0.08, -5.8 % | I > C (p=.03) |
|  | **3) Femoral neck vBMD** (g/cm3) | 0.36 (0.02) | 8mo: 0.02*, 5.6 % | 0.32 (0.01) | 8mo: 0.02, -6.3 % | I > C (p=.02) |
| **Van den Berg-Emons 1998** |  | **I: Physical training program** | | **C: No program** | |  |
| 9 mo | **1) Level of daily physical activity†** | 1.34 (0.25) | 2mo: -0.03  9 mo: 0.21, +16% | 1.24 (0.21) | 2 mo: 0.10  9 mo: 0.10 | NS |
|  | **2) Fat mass** (kg) | 8.1 (6.2) | 2 mo: decreased 9 mo: no changes | 5.7 (2.2) | 2 mo: increased* 9 mo: +1.1 ( SD 1.6) | I > C (p<0.05) |
|  | **3) Peak aerobic power** Watt per kg fat-free mass (FFM) | 0.91 (0.83) | 2 mo: 0.11  9 mo: 0.32*, 35%, range -9 to 376,  12 mo: 0.11, -17%* | 1.11 ( 0.96) | 2 mo: -0.10  9 mo: 0.06  12 mo: 0.04 | 9mo: I > C (p=.05) |
|  | **4) Peak anaerobic power**  (watt per kg FFM) | 2.16 (1.94) | 2 mo: 0.16  9 mo: 0.32, +15%  12 mo: 0.25 | 2.35 (1.75) | 2 mo: -0.09  9 mo: 0.25, +11%* | NS |
|  | **5) Mean aerobic power** (watt per kg FFM) | 1.77 (1.58) | 2 mo: -0.01  9 mo: 0.20, +11% 12 mo: 0.13 | 1.92 (1.45) | 2 mo: -0.04  9 mo: 0.25, +13%* 12 mo: 0.2 | NS |
| ***Constraint induced therapy*** | | | | | | |
| **Charles 2006** |  | **I: CI-therapy with a sling** | | **C: No therapy** | |  |
| 1 wk (1mo, 6mo) | **1) Jebsen-Taylor Test of Hand Function** (modified, max time to complete tasks 720 seconds) | 361.2 (205.4) | 1wk: -82.7 1mo: -92.6 6mo: -88.7 | 314.2 (177.5) | 1wk -13.2 1mo: -53.9 6mo: -17.2 | 1wk: I > C (effect size 0.3, p<.01) 1mo and 6mo: NS |
|  | **2) BOTMP** (subtest 8: speed and dexterity) | 4.8 (3.0) | 1wk: +2.4 1mo: +2.8 6mo: +2.1 | 4.8 (3.7) | 1wk: +0.4 1mo: +0.7 6mo: +1.5 | 1wk: I > C (effect size 0.4, p<.005) 1mo and 6mo: NS |
|  | **3) Caregiver Functional Use Survey** (14 items, 6-point likert scale) | | | | | |
|  | - How frequently | 2.6 (0.7) | 1wk: +0.4 1mo: +0.7 6mo: +0.7 | 2.6 (0.6) | 1wk: -0.3 1mo: -0.1 6mo: 0 | 1wk: I > C (effect size 0.3, p<.01) 1mo and 6mo: NA |
|  | - How well | 2.0 (0.5) | 1wk: +0.5 1mo: +1 6mo: +0.9 | 2.2 (0.5) | 1wk: +0.2 1mo: +0.1 6mo: +0.1 | 1wk: NS 1mo and 6 mo: I > C (Effect size 0.2, p<.01) |
|  | **4) Sensibility** (two point discrimination) | 7.5 (3.1) | 1wk: -0.9 1mo: -1 6mo: +0.1 | 5.7 (3.2) | 1wk: -1.3 1mo: -1.1 6mo: 0 | NS |
|  | **5) Hand-grip force** (hand-held dynamometer) | 2.1 (2.0) | 1wk: -0.1 1mo: -0.2 6mo: +0.3 | 2.2. (2.4) | 1wk: -0.1 1mo: +0.7 6mo: -0.4 | NS |
|  | **6) MAS** |  |  |  |  |  |
|  | - Shoulder | 0.5 (0.5) | 1wk: -0.4 1mo: -0.1 6mo: -0.3 | 0.9 (0.8) | 1wk: 0 1mo: -0.2  6mo: -0.1 | NS |
|  | - Elbow | 1.3 (0.6) | 1wk: -0.2 1mo: -0.1 6mo: -0.2 | 1.3 (1.0) | 1wk: -0.2 1mo: -0.2 6mo: -0.1 | NS |
|  | - Wrist | 1.2 (0.6) | 1wk: 0 1mo: +0.1  6mo: 0 | 1.1 (1.0) | 1wk: +0.4 1mo: +0.3 6mo: +0.5 | NS |
| **Taub 2004** |  | **I: Constraint-induced therapy** | | **C: Early intervention program** | |  |
| 3 wk (3wk, 3mo, 6mo) | **1) QUEST** | No data |  | No data |  | NS |
|  | **1) Emerging Behaviors Scale** (scale 0-31, number of new behaviors emerging) | 12.2 (5.64) | 3wk: 9.3 (range 7-12) | 12.7 (6.5) | 3wk: 2.3 (range 0-6) | I > C (p<.0001) |
|  | **2) Toddler Arm Use Test** (22 tasks, 2wo raters, 4 scales: arm selection (R/L), amount of participation (0-2), how well 0-5, willingness (0-3), global rating 0-10. | | | | | |
|  | - Increased first time use of the more impairment arm | No data | 53.9% improved (SD 35.64) | No data | 18% improved (SD 31.12) | NA |
|  | - Overall independent functional use of the more-impaired arm | No data | 16.8% improved (SD 21.53) | No data | 5% improved (SD 15.4) | NA |
|  | **3) Pediatric Motor Activity Log** (22 items, scale 0-5) | | | | | |
|  | - Amount of use: "how often" | 0.8 (0.44) | post treatment: 2.0  3wk: 1.8  3mo: 1.3  6mo: 1.6 | 1.1 (0.75) | post treatment: 0.1  3wk: 0.1 | I > C (p<.0001) |
|  | - Quality of use: "how well" | 0.9 (0.62) | post treatment: 1.8  3wk: 1.7  3mo: 1.7  6mo: 1.8 | 1.6 (1.2) | post treatment: 0.3  3wk: 0.2 | I > C (p<.0001) |
|  | - Overall score |  |  |  |  | I > C (p<.0001) |
| **Bumin 2001** |  | **I: SPM training individually** | | **C1: SPM training in groups** | |  |
| 3 mo | **1) ASCSIT** | | | | | |
|  | - Double tactile stimuli perception | 29.13 (3.58) | -2.50 (3.31) |  | -1.50 (2.34) | NA |
|  | - Localization of tactile stimuli, total score | 22.71 (7.94) | 6.77 (4.73) |  | 5.48 (6.09) | NA |
|  | - Graphestesia, total score | 9.13 (5.21) | -3.38 (2.03) |  | -3.13 (1.50) | NA |
|  | - Kinesthesia, total score | 51.51 (17.77) | -17.72 (13.75) |  | -6.04 (11.64) | NA |
|  | - Finger identification | 13.69 (2.82) | -1.19 (1.64) |  | -2.63 (3.42) | NA |
|  | - Manual form perception | 9.88 (0.50) | -0.13 (0.50) |  | -0.19 (0.54) | NA |
|  | - Design copying | 2.75 (5.13) | -2.13 (1.71) |  | -2.19 (2.10) | NA |
|  | - Position in space | 8.56 (5.46) | -1.81 (1.22) |  | -2.19 (2.90) | NA |
|  | - Imitation of posture | 2.81 (6.72) | -2.44 (2.06) |  | -3.06 (1.48) | NA |
|  | - Motor accuracy | 96.31 (31.64) | 10.15 (17.24) |  | 14.63 (15.07) | NA |
|  | - Right-left discrimination | 10.69 (5.67) | -2.94 (3.30) |  | -1.69 (2.00) | NA |
|  | **2) Physical Ability Test** | 90.50 (26.30) | -11.25 (24.30) |  | -3.94 (3.55) | NA |
|  |  | **C2: Home program** | |  | | |
|  | **1) ASCSIT** |  |  |  |  |  |
|  | - Double tactile stimuli perception | 29.56 (5.27) | -0.78 (1.20) |  |  |  |
|  | - Localization of tactile stimuli, total score | 28.39 (13.79) | -1.83 (4.49) |  |  |  |
|  | - Graphestesia, total score | 7.78 (6.92) | -0.44 (0.53) |  |  |  |
|  | - Kinesthesia, total score | 58.78 (22.70) | 4.24 (9.60) |  |  |  |
|  | - Finger identification | 12.78 (3.49) | -0.89 (0.78) |  |  |  |
|  | - Manual form perception | 9.22 (1.20) | -0.11 (0.33) |  |  |  |
|  | - Design copying | 2.56 (3.40) | -0.11 (0.33) |  |  |  |
|  | - Position in space | 8.67 (7.92) | 0.00 (0.71) |  |  |  |
|  | - Imitation of posture | 9.22 (7.24) | -0.67 (0.87) |  |  |  |
|  | - Motor accuracy | 78.54 (43.39) | -10.37 (33.21) |  |  |  |
|  | - Right-left discrimination | 9.78 (4.76) | 0.22 (1.79) |  |  |  |
|  | **2) Physical Ability Test** | 95.33 (14.20) | -2.44 (1.33) |  |  |  |
| ***Balance* training** | | | | | | |
| **Ledebt, 2005** |  | **I: Balance training** | | **C: No training** | |  |
| 6-7 wk (10 wk) | **1) Quiet stance on force plate** (time, maximum amplitudes) | | | | | |
|  | - Time on target during quiet standing | No data | No data | No data | No data | NS |
|  | - Displacement in the forward direction | No data | No data | No data | No data | I > C (p=.01) |
|  | - Displacement in the backward direction | No data | No data | No data | No data | I > C (p=.006) |
|  | - Displacement toward the paretic side | No data | No data | No data | No data | NS |
|  | - Displacement toward the non-paretic side | No data | No data | No data | No data | NS |
|  | **2) Dynamic stance on force plate** (maximum amplitudes of COP displacement) | | | | | |
|  | - Leaning forward | No data | No data | No data | No data | I > C (p=.003) |
|  | - Leaning backward | No data | No data | No data | No data | I > C (p<.001) |
|  | - Leaning toward the paretic side | No data | No data | No data | No data | I > C (p=.022) |
|  | - Leaning toward the non-paretic side | No data | No data | No data | No data | I > C (p<.001) |
|  | **3) Step length** (cm)‡ | | | | | |
|  | - Paretic leg | No data | No data | No data | No data | NS |
|  | - Non-paretic leg | No data | No data | No data | No data | I > C (p=.017) |
|  | **4) Step length asymmetry** (percentage of the average step length in forward swinging of both legs) | No data | No data | No data | No data | NA |
| ***Therapy with animals*** | | | | | | |
| **Benda 2003** |  | **I: Equine-assisted therapy (hippo therapy)** | | **C: Stationary barrel** | |  |
| 8 min | **1) Muscle asymmetry with EMG**§ | No data | 8min: 55.5 (82.5), 64.6% (28.3) | No data | 8min: 11.9 (29.9), 12.8% (88.8) | NS |
| **MacKinnon 1995** |  | **I: Horseback riding, moderate** | | **C1: No hippo therapy, moderate** | |  |
| 6 mo | **1) GMFM** (scale 0-100) |  |  |  |  |  |
|  | - Sitting | No data | -0.40 | No data | 2.00 | NS |
|  | - Standing | No data | 0.40 | No data | -0.50 | NS |
|  | - Walking | No data | 0.40 | No data | 0.00 | NS |
|  | - Total score | No data | 0.40 | No data | 1.50 | NS |
|  | **2) Bertoti** (posture measured in sitting position) | No data | 1.20 | No data | 1.00 | NS |
|  | **3) BOTMP**  **-** fine motor, item 6 | No data | No data | No data | No data | NS |
|  | - Fine motor, item 8 | No data | No data | No data | No data | NS |
|  | **4) PDMS-FM** | No data |  | No data |  |  |
|  | - Grasping | No data | 1.80 | No data | -0.50 | I > C1 (p=.045) |
|  | - Hand use | No data | 3.40 | No data | 1.25 | NS |
|  | - Eye-hand coordination | No data | 4.20 | No data | 4.50 | NS |
|  | - Manual dexterity | No data | 1.00 | No data | 2.25 | NS |
|  | - Total score | No data | 9.80 | No data | 8.50 | NS |
|  | **5) VABS -** ADL | No data | -18.73 | No data | -19.33 | NS |
|  | - Socialization | No data | -3.00 | No data | -5.75 | NS |
|  | **6) HSPC** | No data | -3.00 | No data | 2.80 | NS |
|  | **7) CBC** | No data |  | No data |  | NS |
|  | - Activities | No data | -0.70 | No data | 0.42 | NS |
|  | - School | No data | 0.63 | No data | -0.25 | NS |
|  | - Social | No data | -0.90 | No data | 0.13 | NS |
|  | - Total prob | No data | -1.40 | No data | -4.67 | NS |
|  | - Total COMP | No data | -1.73 | No data | -9.50 | NS |
|  |  | **C2: Horseback riding, mild** | | **C3: No hippo therapy, mild** | |  |
|  | **1) GMFM** (scale 0-100) |  |  |  |  |  |
|  | - Sitting | No data | -0.20 | No data | -2.05 | NS |
|  | - Standing | No data | 1.40 | No data | 2.55 | NS |
|  | - Walking | No data | 0.60 | No data | 0.50 | NS |
|  | - Total score | No data | 1.40 | No data | 1.00 | NS |
|  | **2)** Posture measured in sitting position¶ | No data | -0.20 | No data | -1.30 | NS |
|  | **3) BOTMP**  - Fine motor, item 6 | No data | -0.75 | No data | -1.67 | NS |
|  | - Fine motor, item 8 | No data | 0.60 | No data | 5.33 | NS |
|  | **4) PDMS-FM** | No data |  | No data |  |  |
|  | - Grasping | No data | 0.00 | No data | 0.60 | NS |
|  | - Hand use | No data | 0.40 | No data | 0.10 | NS |
|  | - Eye-hand coordination | No data | 2.60 | No data | 2.30 | NS |
|  | - Manual dexterity | No data | 0.20 | No data | 2.05 | NS |
|  | - Total score | No data | 3.20 | No data | 5.05 | NS |
|  | **5) VABS**: - ADL | No data | -1.80 | No data | -0.45 | NS |
|  | - Socialization | No data | -2.80 | No data | -5.40 | NS |
|  | **6) HSPC** | No data | 2.20 | No data | 3.55 | NS |
|  | **7) CBC** | No data |  | No data |  | NS |
|  | - Activities | No data | 1.00 | No data | -0.62 | NS |
|  | - School | No data | 0.00 | No data | -0.25 | NS |
|  | - Social | No data | 0.30 | No data | -0.97 | NS |
|  | - Total prob | No data | 3.20 | No data | 2.40 | NS |
|  | - Total COMP | No data | -7.0 | No data | -10.0 | NS |

* Statistically significant difference to the baseline values in the within group analysis,

**†** total energy expenditure/sleeping metabolic rate or total energy expenditure/resting metabolic rate,

‡ distance of 2 successive foot contacts when swinging forward, calculated from the displacement of the COP along the progressive axis during walking,

§ asymmetry score for the muscle group most affected during the pretest activity in sitting, standing or walking, and the mean change in percentage (pre-test asymmetry score/post-test asymmetry score x 100). 16 surface electrodes (posterior cervical, posterior thoracic, posterior lumbar, adductor and abductor muscle groups of upper thigh) connected to 2 transmitters. Absolute differences in mean microvolt readings between left and right-side individual muscle groups were calculated during sitting, standing and walking and recorded as asymmetry scores. The highest pre-test asymmetry score for the most affected muscle group for each child was used and compared with post-test value and converted into a percentage score.

¶ by a scale developed by Bertoti DB: Therapeutic riding conferences-Positive progress. In Proceedings of the 6th International Therapeutic Riding Congress; August 23-27; Toronto, Ontario. 1988: 400-405.

wk=week/s, mo=month/s, SD=standard deviation, SEM=standard error of mean, NDT=neurodevelopmental therapy, PT=physiotherapy, SPM=sensory perceptual motor, CI=confidence interval, NS= no statistically significant difference between the groups, I > C=Statistically significant difference in favor of the intervention group, C > I =Statistically significant difference in favor of the control group.

ASCSIT=Ayres Southern California Sensory Integration Test, BMC=Bone mineral density, vBMD = Volumetric bone mineral density, BOTMP=Bruininks-Oseretsky Test of Motor Proficiency, CHQ=Child health Questionnaire, CBC=Child Behavior Checklists, EMG=Electromyography, GMDS=Griffith's Mental Developmental Scales, GMFM = Gross Motor Function Measure, GMPM=Gross Motor Performance Measure, HOME=Home Observation for Measurement of the Environment, HSPC=Harter Self-perception Profile for Children, MAS=Modified Asworth Scale, MPOC=Measure of Processes of Care, PCI=Physiological Cost Index, PEDI=Pediatric Evaluation of Disability Inventory, PDMS-FM= Peabody Developmental Motor Scales Fine Motor, QUEST=Quality of Upper Extremity Skills Test, ROM=range of motion, VABS=Vineland Adaptive Behavior Scale.
